# 4-dimensional functional profiling in the convulsant-treated larval zebrafish brain

**DOI:** 10.1038/s41598-017-06646-6

**Published:** 2017-07-26

**Authors:** Matthew J. Winter, Dylan Windell, Jeremy Metz, Peter Matthews, Joe Pinion, Jonathan T. Brown, Malcolm J. Hetheridge, Jonathan S. Ball, Stewart F. Owen, Will S. Redfern, Julian Moger, Andrew D. Randall, Charles R. Tyler

**Affiliations:** 1 Biosciences, College of Life and Environmental Sciences, Exeter, Devon EX4 4QD United Kingdom; 2 Medical School, University of Exeter, Exeter, Devon EX4 4PS United Kingdom; 3 AstraZeneca, Global Compliance, Alderley Park, Macclesfield, Cheshire SK10 4TF United Kingdom; 4 AstraZeneca R&D Innovative Medicines, Drug Safety & Metabolism, Babraham Research Campus, Cambridge, CB22 3AT United Kingdom; 5 Physics and Medical Imaging, College of Engineering, Mathematics and Physical Sciences, University of Exeter, Exeter, Devon EX4 4QL United Kingdom

## Abstract

Functional neuroimaging, using genetically-encoded Ca^2+^ sensors in larval zebrafish, offers a powerful combination of high spatiotemporal resolution and higher vertebrate relevance for quantitative neuropharmacological profiling. Here we use zebrafish larvae with pan-neuronal expression of GCaMP6s, combined with light sheet microscopy and a novel image processing pipeline, for the 4D profiling of chemoconvulsant action in multiple brain regions. In untreated larvae, regions associated with autonomic functionality, sensory processing and stress-responsiveness, consistently exhibited elevated spontaneous activity. The application of drugs targeting different convulsant mechanisms (4-Aminopyridine, Pentylenetetrazole, Pilocarpine and Strychnine) resulted in distinct spatiotemporal patterns of activity. These activity patterns showed some interesting parallels with what is known of the distribution of their respective molecular targets, but crucially also revealed system-wide neural circuit responses to stimulation or suppression. Drug concentration-response curves of neural activity were identified in a number of anatomically-defined zebrafish brain regions, and *in vivo* larval electrophysiology, also conducted in 4dpf larvae, provided additional measures of neural activity. Our quantification of network-wide chemoconvulsant drug activity in the whole zebrafish brain illustrates the power of this approach for neuropharmacological profiling in applications ranging from accelerating studies of drug safety and efficacy, to identifying pharmacologically-altered networks in zebrafish models of human neurological disorders.

## Introduction

Real-time cell-level imaging of neural activity is a powerful means to study the activity of neuronal assemblies at scales ranging from the sub-cellular to entire nervous systems. The most widely employed imaging-based readouts for this are fluorescent reporters of intracellular Ca^2+^ concentration. These effectively reflect neural activity as voltage changes in neurons are usually coupled closely with Ca^2+^ transients that arise through the gating of voltage-sensitive Ca^2+^ channels. Of the fluorophores available, GCaMPs, which can be genetically encoded, are amongst the most sensitive^1^; Ca^2+^ binding to the GCaMP molecule results in conformational changes that provide a fluorescent indication of increased neural activity^2^. GCaMPs have been employed in a range of model organisms^3^; amongst these, larval zebrafish offer an unparalleled combination of higher vertebrate (including human) relevance, optical transparency, and a small brain size (comprising approximately 100000 neurons)^4^ allowing simultaneous spatial coverage of multiple brain regions within a fully intact connectome. Moreover, the availability of transgenic zebrafish, in which genetically-encoded GCaMPs are under the control of the pan-neuronal *elavl3* promotor have greatly facilitated the potential for assessing neural network dynamics (e.g. refs 5–7). When coupled with imaging modalities with high spatiotemporal resolution, such as light sheet microscopy (LSM)^8^, such models offer a uniquely powerful experimental system with which to functionally characterise large-scale, integrated neuronal assemblies at rest^4^, under conditions of sensory stimulation^9, 10^ or, as in the case with the current study, following treatment with neuroactive drugs.

Using four exemplar drugs known to induce neural network hyperactivity by different molecular mechanisms in both mammals and zebrafish, we utilise a combination of LSM and a transgenic zebrafish (*elavl3*:*GCaMP6s*)^11^ to quantify functional brain responses in 4 dimensions. Our approach addresses certain key challenges associated with profiling drug action in a living vertebrate, such as the contextualised quantification of pharmacologically-modified neural function within a 3D landscape of brain anatomy^4^. Accordingly, we present a computational image analysis framework that enables 3D anatomical registration against a standardised larval zebrafish brain, thus providing quantitative functional data for defined regions across the whole larval brain. Using this methodology we have quantified several measures of neural activity in multiple brain regions, revealing differential spatiotemporal patterns associated with differing molecular mechanisms of drug action. The use of *in vivo* extracellular local field potential recording from the optic tectum of comparable animals provides additional information on the temporal profile characteristics of drug-induced neural activity to aid interpretation of the GCaMP imaging data.

Here we present novel data that validate the use of this experimental system for the routine 4D functional profiling of drug action in the brain of a highly relevant vertebrate model. Furthermore our approach has many potential applications ranging from studies of CNS drug safety and efficacy, and, when combined with the relative simplicity of gene editing in the zebrafish, to the identification of functionally-altered network activity in various human neurological disorders.

## Materials and Methods

### Experimental animals

Transgenic *elavl3* *:GCaMP6s*, in an unpigmented Casper background, were the kind gift of Misha B. Ahrens (Janelia Research Campus, Howard Hughes Medical Institute, Ashburn, Virginia, USA) and were used at 4 days post fertilization (dpf). This line incorporates stable cytoplasmic expression of GCaMP6s under the control of the *elavl3* promotor^11^. All animal work was approved by the University of Exeter’s Animal Welfare and Ethical Review Body, and undertaken under project and personnel licences granted by the UK Home Office under the United Kingdom Animals (Scientific Procedures) Act, and in accordance with The University of Exeter’s ethical policies.

### Test compounds

The drugs selected for study (Table 1) were: pentylenetetrazole (PTZ), a GABA_A_ receptor antagonist^12^; pilocarpine, a muscarinic acetylcholine receptor (mAChR) agonist^13^; strychnine, a chemoconvulsant that acts predominantly via glycine receptor (GlyR) antagonism^14^; and 4-Aminopyridine (4AP), a K^+^ channel blocker that enhances neuronal excitability and consequently the release of various neurotransmitters including glutamate^15, 16^.

**Table 1:** Compounds and respective exposure regimes used for imaging neural activity responses in 4dpf *elavl3*:*GCaMP6s* zebrafish larvae.

Compound	CAS No.	Drug concentrations in external medium	Pre-imaging exposure period
4-Aminopyridine (4AP)	504-24-5	1, 0.5 and 0.25 mM	15 minutes
Pentylenetetrazole (PTZ)	54-95-5	5, 2.5 and 1.25 mM	20 minutes
Pilocarpine hydrochloride	54-71-7	5, 2.5 and 1.25 mM	30 minutes
Strychnine hemisulphate (STR)	60-41-3	200, 100 and 50 µM	20 minutes

All sourced from Sigma-Aldrich, Poole, UK.

### Drug Treatment and Light Sheet Microscopy

**Figure 1:**
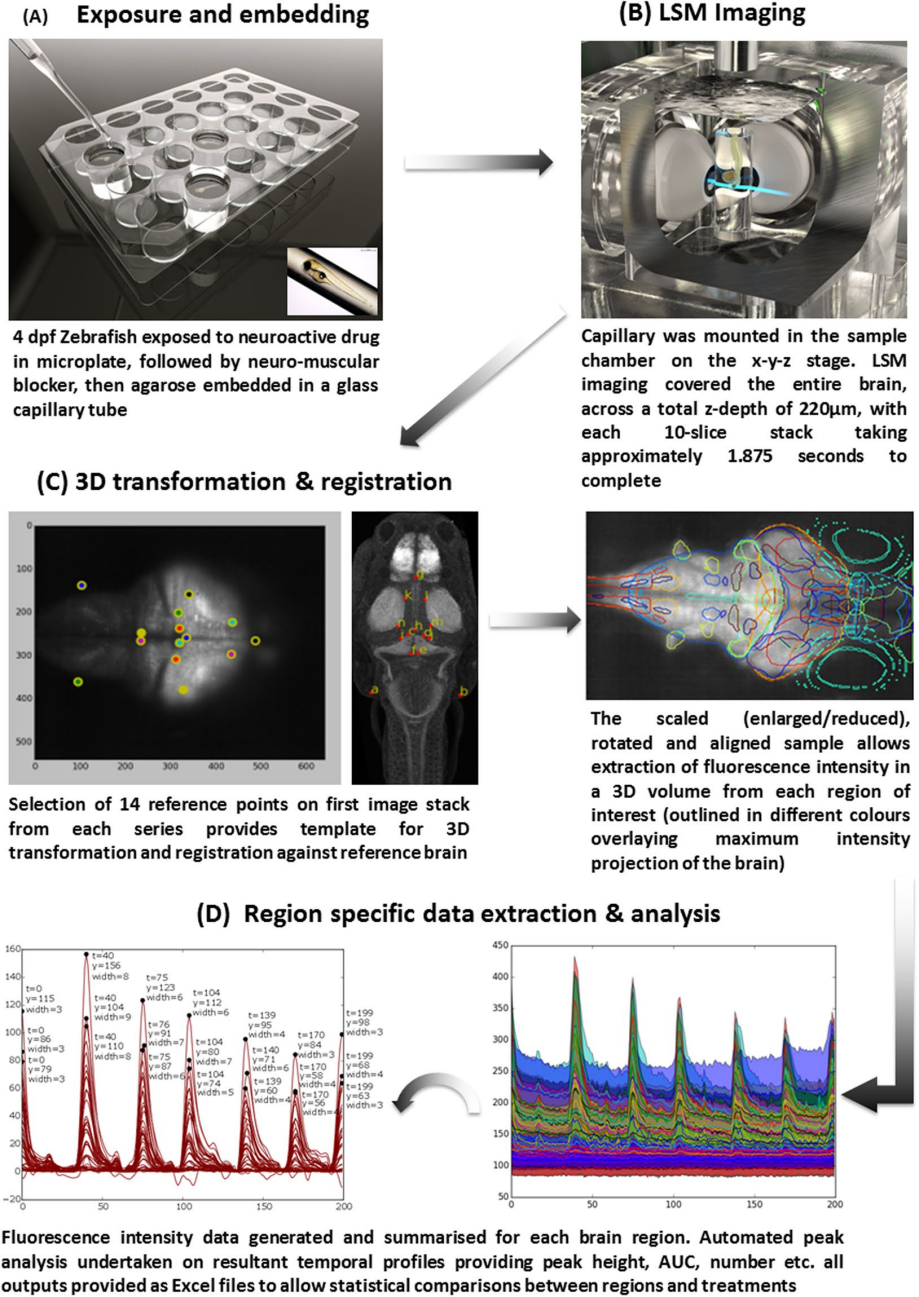
Schematic of the basic experimental process used to obtain 4D whole brain neural activity data from drug treated larval *elavl3*:*GCaMP6s* zebrafish (**A**) Larvae are cultured to 4 days post fertilisation (dpf), exposed to each drug, and neuromuscular blocker and embedded in agarose in a capillary tube; (**B**) larvae are then imaged on the LSM to capture the whole brain volume; (**C**) Resultant images are processed using a custom Python script in which 3D anatomical registration against a standard brain allows identification of regions of interest (ROIs); (**D**) fluorescence intensity data are summarised for ROI and automated peak analysis performed on the resultant temporal profiles (example plots that are automatically output are shown for illustrative purposes).

A schematic of the basic experimental process is presented in Fig. 1. Larvae were pre-exposed to each drug in a well of a 24 well plate (Table 1), and then transferred to another well containing drug plus the anti-nicotinic neuromuscular blocker tubocurarine (4 mM), until muscle tone was lost. Each drug was tested at 3 concentrations alongside an untreated control group (8 larvae per treatment). The concentrations and exposure periods were selected based on our previous studies in which they were observed to induce convulsions, but were well below lethal concentrations in larval zebrafish^17^ (Supplementary Table 1), and also from range-finding work undertaken using wide-field fluorescence microscopy (see below). Following drug exposure, individuals were transferred to drug plus tubocurarine in 1.4% Low Melting Point (LMP) agarose, and drawn into a clear borosilicate glass capillary (940 µM internal diameter) plugged with 1.5% LMP agarose. Control larvae were treated identically without the presence of the chemoconvulsant. Each mounted fish was positioned vertically (Head down), and optical sectioning was undertaken repeatedly in the horizontal plane from the dorsal to ventral surface of the larva. This was repeated for 6 mins generating 200 × 10-Z-plane image stacks, each spanning a total depth of 220 μm. At the end of each 6 min run, the larva was checked for a normal heart rate and blood flow by microscopy to ensure viability. Standard wide-field imaging (see below) suggested minimal photobleaching (<10%/hour) supporting the ability to undertake longer term assessment. However, the image capture parameters employed were considered appropriate for obtaining a representative period of drug-induced neural activity across all major brain structures, whilst yielding datasets suitable for routine image processing and analysis techniques.

The LSM (See Supplementary Figure 1) was custom built using the Open Access platform for SPIM^8, 18^. The system comprised a 488 nm Argon laser (Melles Griot, Didam, Netherlands) for illumination, a 20x/0.5 NA objective lens (Olympus, Southend-on-Sea, UK) with an intermediary magnification of 1x, followed by a 525/50 nm emission filter and 495 nm bandpass filter (Chroma, Olching, Germany) to collect emitted light, and a 5.5MP Zyla sCMOS camera (30FPS, 640 × 540 pixels, 4 × 4 binning, 40 ms exposure, Andor, Belfast, Northern Ireland) to capture images. The camera and rotational axis (Picard Instruments, Albion, USA) were controlled though μManager^19^ and the OpenSPIM plugin^18^. The Point Spread Function (PSF) of our system was determined by performing Z-stacks of 50 × 0.5 µm fluorescent beads, embedded in 1% agarose within a glass capillary. Images were acquired at z-intervals of 1.5 µm using the same system settings employed during data acquisition (detailed above), and the PSF calculated using the MOSAICsuite PSF Tool (http://imagej.net/MOSAICsuite). The spatial resolution of our system was measured using a segment from a multi-grid stage micrometer (Edmund Optics, UK) with 100 μm divisions. The grid was imaged in the sample chamber using the same optics and medium to determine the pixel length of each 100 μm division. Each 100 μm division at 1 × 1 binning was 304 pixels, and at 4 × 4 binning was 76 pixels.

### Image processing and analysis

**Figure 2:**
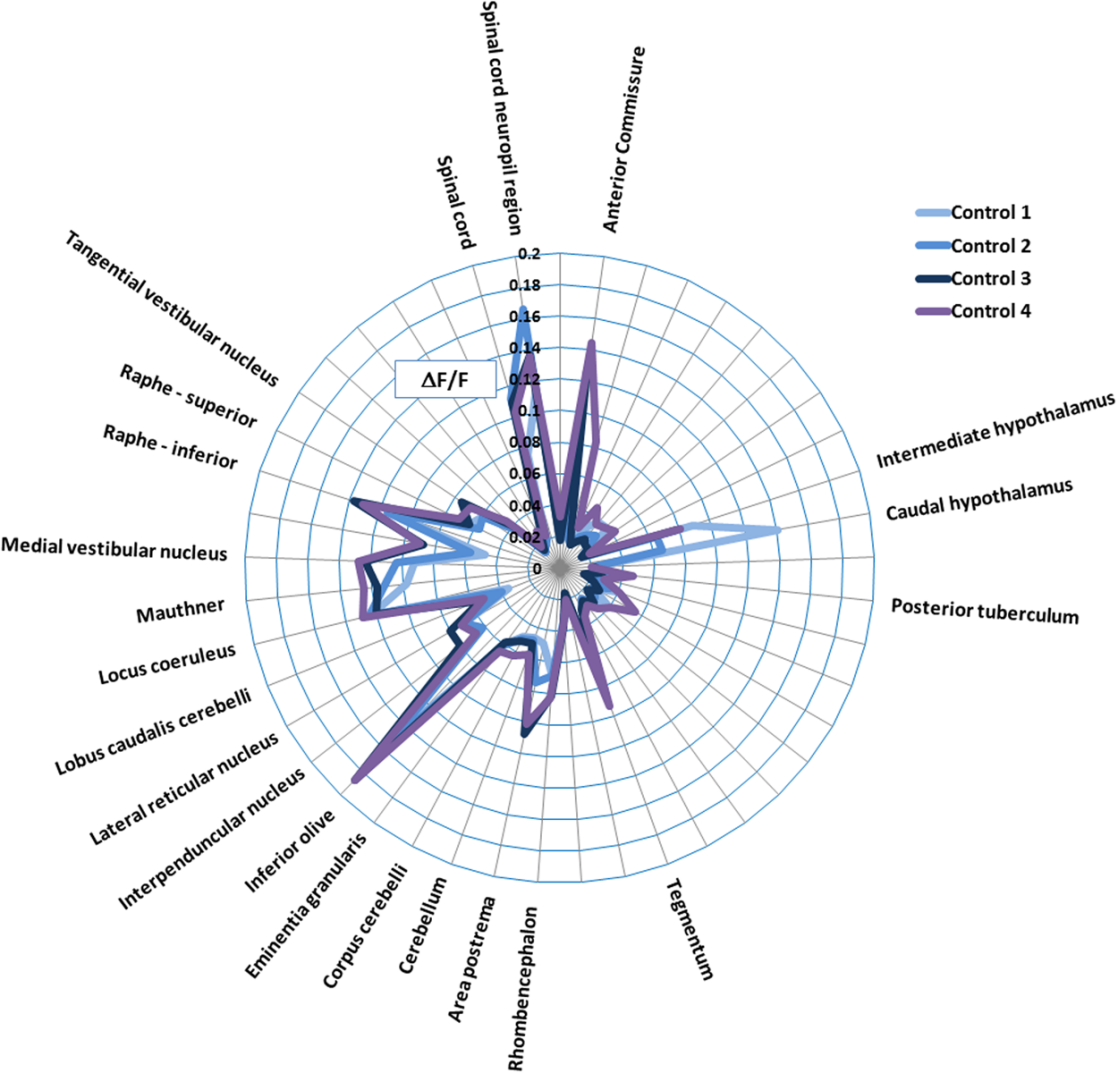
Summary of the control fish data across 4 experiments. Data are shown as the average of ΔF/F ( = (F_1_ − F_0_)/F_0_*100, where F_1_ = peak fluorescence intensity, and F_0_ = baseline fluorescence intensity) of the fish within each control group. Only those regions showing activity greater than the median of all areas (>0.0386) are labelled. Note the consistency in brain regions showing measurable baseline activity. In particular, activity was most pronounced in the anterior commissure, hypothalamus, tegmentum, rhombencephalon, inferior olive, locus coeruleus, mauthner cells, medial vestibular nucleus, superior and inferior raphe and spinal cord neuropil region.

Data processing was performed using a custom Python image processing pipeline incorporating functions from the Scikit-image^20^, Scipy^21^ and Scikit-Learn^22^ libraries. Each 3D image was first shift-corrected using 3D registration and then down-sampled by averaging across 3 × 3 × 1 voxel blocks. The data were then baseline corrected by subtracting each voxel’s baseline, estimated using a sliding minimum filter. Due to the absence of an appropriate control with which to normalise to account for regional variations in the expression of *elavl3*:*GCaMP6s*, the data presented in Fig. 2 (see later) were then divided by the same baseline. Next, a labelled reference brain (http://engertlab.fas.harvard.edu/Z-Brain/downloads.html)^23^ was aligned with each image in 3D by using key-point registration allowing full 3D affine transforms to optimise the quality of the alignment. Key-points comprised 14 predetermined anatomical reference points that were user-selected on a representative stack from each series. The optimal affine transformation for aligning the reference points to the expert-selected points was then determined using a standard optimisation algorithm (Umeyama). The whole series was then aligned to the labelled reference brain using this optimal transform, and registration was undertaken for 45 anatomical Regions of Interest (ROIs) selected to encompass major brain structures (see Supplementary Table 2). Each dataset was visually inspected for accuracy of the registration process using the reference brain overlay image shown in Fig. 1, panel C, and any showing poor alignment were reprocessed following adjustment of the user defined key-points. The error of the registration process was estimated by calculating the median distance between the aligned reference key-points and the user selected key-points for each dataset.

For each ROI, fluorescence intensity data were provided as the time-averaged mean, median and standard deviation of the incorporated voxels. The median is a robust measure of the central tendency of the fluorescence signal within each ROI as it is less influenced by small variations in the spatial accuracy of the registration process. In addition, temporal profile peak analyses were undertaken for each ROI. For this, all activity profile baselines underwent fine-scale smoothing using Gaussian filtering (sigma = 1.5) to preserve the signal features while smoothing high-frequency noise in the signal. For peak analysis, the baseline from the smoothed signal was subtracted (as above), and then a threshold applied of twice the standard deviation to identify significant peak regions. Any peaks spanning 2 or more consecutive imaging cycles (>1.875 secs, and thus considered non artefactual) were automatically logged and analysed, and for each animal mean peak height, peak width, peak separation in frames, Area Under the Curve (AUC) from summed intensities, and number of peaks were provided in an output file.

The use of an independent sample experimental design meant that the most appropriate representation of untreated baseline activity was the average of the median intensity values from the water-exposed animals, within each region. Consequently, statistical analysis of concentration-response relationships within each ROI was undertaken using median fluorescence intensity data comparing treated with untreated groups. This was achieved using Kruskal-Wallis tests coupled with Dunn’s post hoc comparisons of treated with corresponding non-treated control fish data. The four control groups were also compared within each ROI using the same method.

For graphical representation of relative induction or suppression of neural activity between compounds and brain regions, data are expressed as the % increase over the same region from the mean of the corresponding untreated control group according to the formula: ΔF/F = (F_1_ − F_0_)/F_0_*100, where F_1_ = treated group fluorescence intensity, and F_0_ = control group fluorescence intensity.

### Wide-field fluorescence microscopy for range finding

Full details of the wide-field fluorescence microscopy are contained within the Supplementary Materials and Methods section. Briefly, larvae were immobilized with tubocurarine, mounted in custom designed microchambers in LMP agarose, and transferred onto a standard inverted microscope equipped with a 10x objective, fast switching fluorescent light source and sensitive high speed CCD camera. Excitation was provided by constant 490 nm illumination, and images collected and assessed. In each case, a drug-free baseline recording of 10 mins was undertaken prior to the addition of the test compound to the microchamber, and subsequent recording was continued for a further 70 mins.

### *In vivo* electrophysiology

Full details are contained in the Supplementary Materials and Methods. Briefly, larvae were immobilised with tubocurarine and then fixed and positioned dorsal side up in the recording chamber in LMP agarose containing extracellular solution (ECS). Under 4x magnification, a glass ECS-filled microelectrode (resistance of 3–5 MΩ) was inserted into the optic tectum (Supplementary Figure 2) to record extracellular local field potential from small networks of neurons. Larvae were equilibrated for 600 secs prior to data acquisition to establish a baseline response. Local field potential (LFP) recording protocols allowed for a 300secs baseline period before drug addition to the ECS and experimental recording lasted for a total of 4200 secs. During all experimentation on larval zebrafish, the heart rate was monitored visually to confirm viability. Comparisons of the frequency (Hz) and power (mV^2^) of neuronal network events were made before (0–900 secs) and after drug addition (3300–4200 secs). Student’s t-tests were performed on paired data with statistical significance defined as P < 0.05.

## Results

### Estimates of registration error, and spatial and temporal resolution

The average of the median error of the automated 3D affine registration processes was calculated as 27.56 ± 0.43 μm (median, ± SEM, n = 128 datasets), a figure in line with the error reported for an alternative registration process published by Ahrens *et al*.^24^.

The spatial resolution of the system is summarised in Supplementary Figure 3. The theoretical point spread function (PSF), calculated from the average of 50 × 0.5 μm fluorescent beads and expressed as the width at half maximum intensity, was 0.751 μm. The measured lateral resolution was 3.04 pixels per μm, or 0.76 pixels per μm when employing the 4 × 4 binning used to achieve faster acquisition rates for registration of broad brain regions.

The temporal resolution of our system was 1.875 secs for each full z-series (including stage movement and image acquisition). This cycle time was suitable for capturing dynamic neural events at a level of spatial resolution appropriate for our intended aim of mapping whole brain functional responses to neuroactive drug treatment.

### Mapping whole brain neural activity in untreated larvae

Control larvae exhibited a relatively low level of neural activity compared with drug-treated animals; however, there was consistent above-baseline activity in certain brain regions (Fig. 2). Active regions were mainly rhombencephalic in origin and included the area postrema (sensory circumventricular organ involved in multiple autonomic processes)^25^, inferior olive (predominantly associated with adaptive motor control)^24^, locus coeruleus (involved in autonomic arousal associated with stress)^26, 27^, Mauthner cells (control of the escape response)^28^, raphe (serotonergic nuclei with functions in nociception, sleep-wake cycles and alertness)^29^, and medial vestibular nucleus (vestibular signal processing)^30^, in addition to the telencephalic anterior commissure (interhemispheric functional connectivity)^31^, diencephalic caudal hypothalamus (multisystem homeostasis)^32^, and mesencephalic tegmentum (motor control)^33, 34^.

### Mapping whole brain neural activity during drug treatment - time averaged intensity data

**Figure 3:**
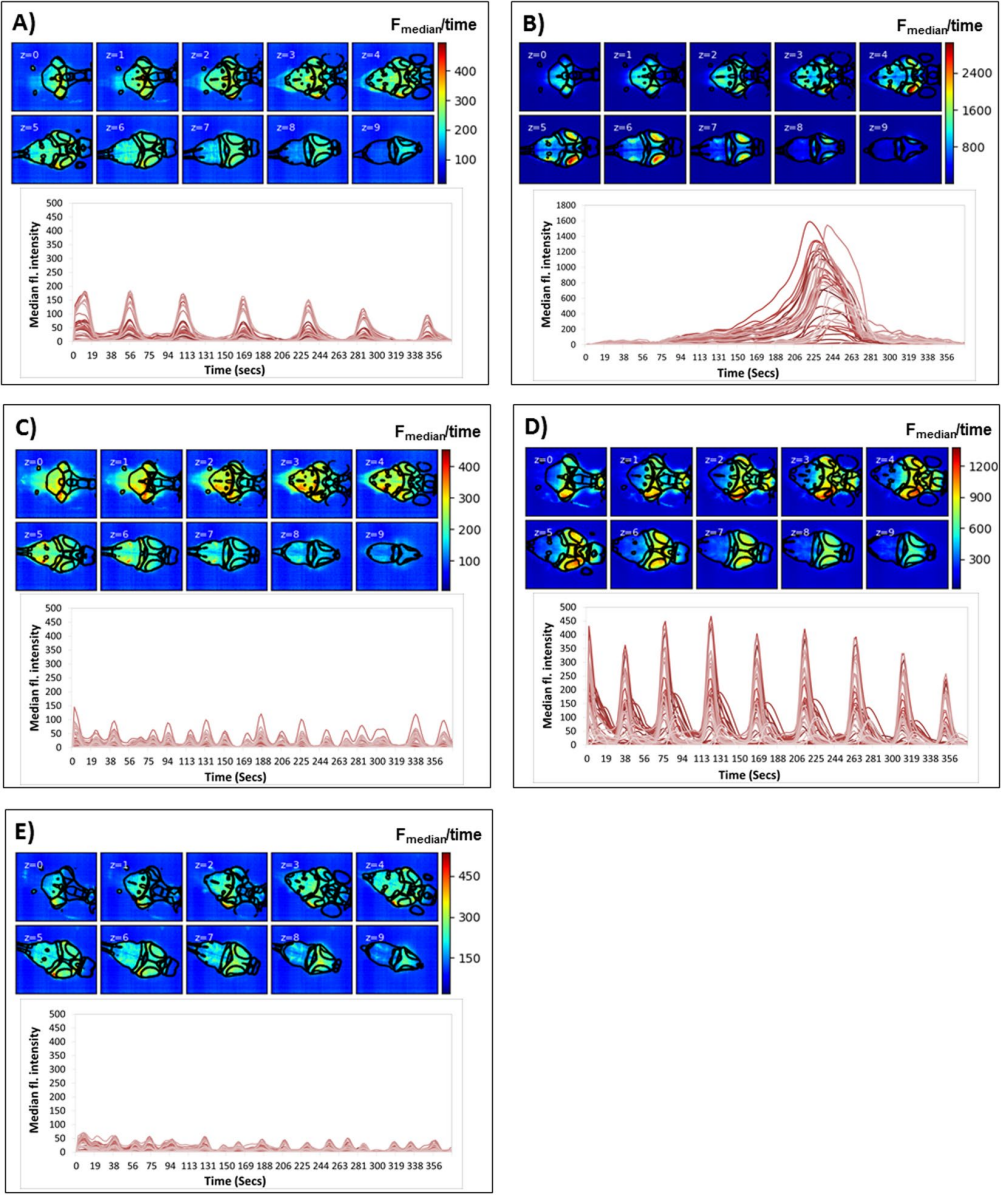
Example larvae exposed to each of the 4 neuroactive drugs plus an untreated control. Images are median intensity projections across time, for each z-plane (0–9 ventral to dorsal surface) and the corresponding temporal profile with each line representing the median intensity of the voxels within each of the 45 3D-registered anatomical regions. Each panel represents an example larva treated with: (**A**) PTZ, (**B**) 4-AP, (**C**) Pilocarpine (**D**) Strychnine and (**E**) untreated control. Superimposed on each image are outlines of the regions registered within that z-plane. From each montage, one can clearly see differences in the GCaMP fluorescence intensity within specific brain regions, and the different characteristics of the temporal profiles generated within these regions of interest. Accompanying colour scales show the fluorescence intensity range for each montage as the median fluorescence intensity value averaged over the experimental duration.

Treatment with the 4 drugs confirmed distinct spatiotemporal patterns of neural activity initially identified using standard wide field fluorescence imaging (Supplementary Figure 4). Using LSM, this differential patterning was evident both in the anatomical ROIs being activated or supressed, as well as in the temporal profile characteristics exhibited. Figure 3 exemplifies the different patterns of neural activity shown for each drug, compared with that exhibited by a typical untreated control animal. From the temporal profiles shown, one can see the pronounced, relatively high amplitude Ca^2+^ transients in the drug-treated larvae compared with the low level undulation of the untreated state. Furthermore, the waveform, peak separation, peak height and number of peaks clearly varies between drugs, in addition to the regions affected, as shown in the pseudo-coloured fluorescence intensity images that accompany these profiles.

**Figure 4:**
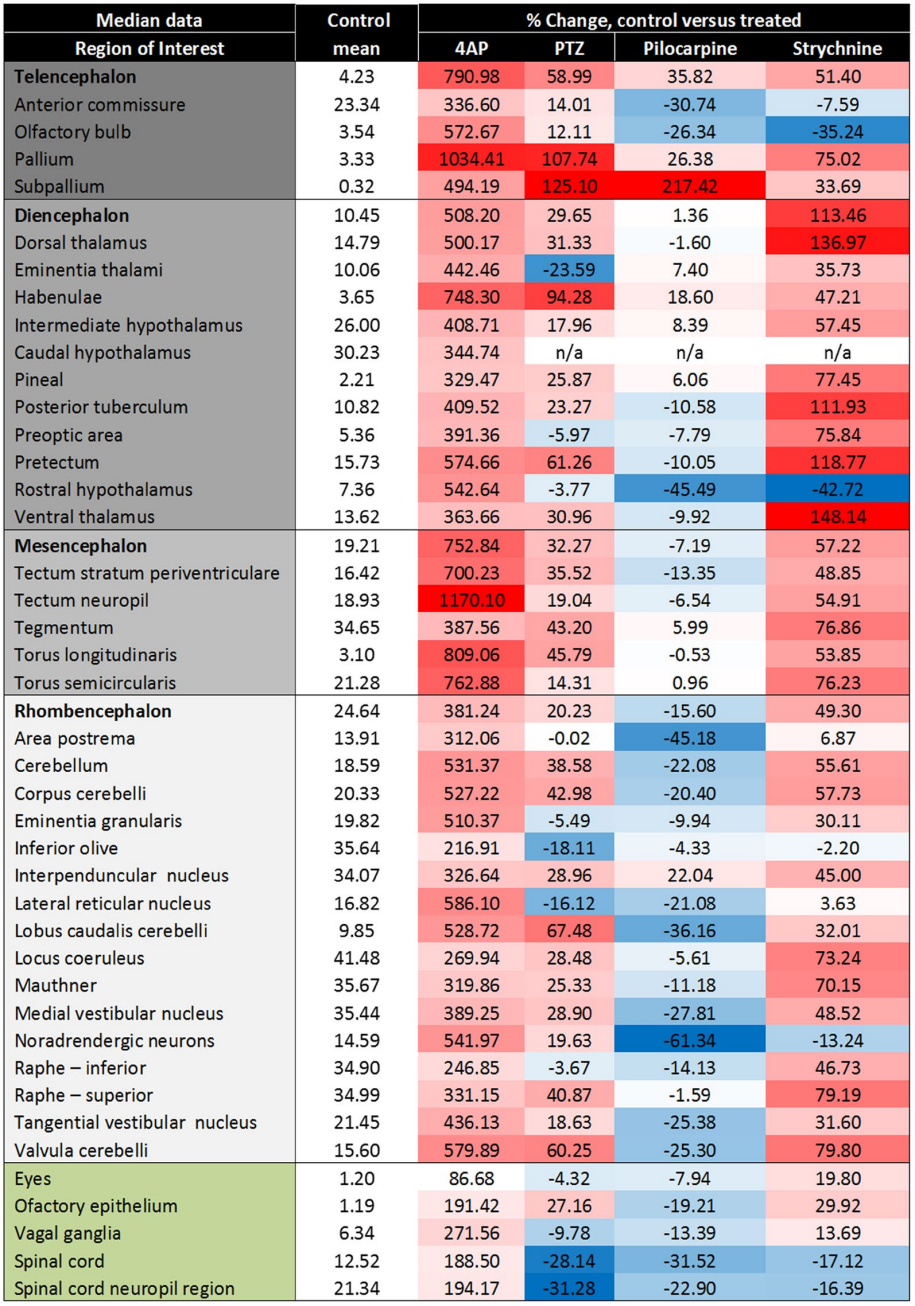
Summary of the voxel GCaMP6s fluorescence intensity obtained per anatomical region for each of the model chemoconvulsant compounds applied. Data are expressed as the time-averaged median fluorescence intensity values, averaged across all fish in that treatment group, expressed the % change versus the corresponding control fish group (second left column presented for brevity as the mean across the 4 control groups): ΔF/F = (F_1_ − F_0_)/F_0_*100 (where F_1_ = treated group fluorescence intensity, F_0_ = control group fluorescence intensity). Colour coding represents the degree of activation (shades of red), or suppression (shades of blue) versus that region in the control larvae, relative to other brain regions within that treatment group. The treatment group shown is that at which the highest activity was observed, which was the top concentration for 4AP, pilocarpine and strychnine, but 2.5 mM (the second highest) for PTZ due to a slight dip in neural activity at the highest treatment level. Colour coding in the left most column represents anatomical categorisation as subdivisions of the telencephalon, diencephalon, mesencephalon and rhombencephalon, along with ganglia (green) respectively from top to bottom. n/a – values not obtained due to a failure in registration probably due to the high z-depth of this region. N = 8 larvae were imaged per group. For corresponding SEM values, see Supplementary Table 3.

For each drug, Fig. 4 summarises the median GCaMP6s fluorescence intensity for each ROI as a % increase compared with the corresponding control group value, thus allowing visual comparison of the relative induction or suppression of each ROI compared to each another, and between drugs. 4AP-treated animals exhibited a widespread and considerable increase in activity across the majority of ROIs. None of these exhibited a reduction in activity and a number (e.g. pallium, habenulae, various tectal sub-regions, and the torus longitudinalis and semicircularis) exhibited average levels of more than 7 times that shown in the same ROI from the mean of the untreated control larvae. Although some of these areas also showed considerable inter-animal variation, (Supplementary Table 3) several ROIs showed a significant and concentration-dependent increase in median fluorescence intensity (Supplementary Table 4), namely the torus semicircularis, Mauthner cells, and the medial and tangential vestibular nuclei (all P < 0.05).

Larvae treated with PTZ showed less widespread and extensive activation compared with the 4AP treated larvae, but with specific ROIs exhibiting a range of increased and decreased % activity compared with the corresponding control animals (Fig. 4). Most notably, there was a greater than 15% reduction in GCaMP fluorescence in the eminentia thalami, inferior olive, lateral reticular nucleus, and the spinal cord and associated neuropil region. Again, inter-animal variability in some cases was high (Supplementary Table 3), and consequently only the eminentia thalami, preoptic area and spinal cord exhibited a significant reduction in activity compared with the corresponding untreated larval data (all P < 0.01). This variability was most evident in terms of the areas showing apparent stimulation of activity of activation as a number of ROIS exhibited a >50% increase in activity compared with control larvae (e.g. pallium and subpallium, habenulae, pretectum, lobus caudalis cerebelli, and valvula cerebelli), although only one achieved statistical significance (Olfactory bulb, P < 0.01, Supplementary Table 4).

When the time averaged GCaMP fluorescence intensity data were considered, pilocarpine treatment resulted in predominantly suppressed neural activity compared with control larvae (Fig. 4). Most notably, >30% reductions in fluorescence intensity compared with the corresponding control data were observed in the anterior commissure, rostral hypothalamus, area postrema, lobus caudalis cerebelli, and the noradrendergic neurons of the interfascicular and vagal areas. As occurred with the other drugs, there was some inter-animal variability (Supplementary Table 3) and consequently only the area postrema (P < 0.05), and the noradrendergic neurons of the interfascicular and vagal areas (P < 0.01) were significantly lower than in controls, in addition to the inferior raphe (P < 0.05) and spinal cord (P < 0.01, Supplementary Table 4). Conspicuous by their increased levels of activity (>18% of controls) were the pallium, subpallium, habenulae, and interpenduncular nucleus, although none achieved statistical significance (Supplementary Table 4).

In strychnine-exposed larvae, there was an apparent altered activity in multiple ROIs compared with the untreated control larvae (Fig. 4), most notably >100% increase over controls in multiple sub-regions of the diencephalon (e.g. the dorsal and ventral thalamus, posterior tuberculum, and pretectum), and >30% decrease in activity in the olfactory bulb and rostral hypothalamus. Despite the widespread trends for altered GCaMP fluorescence in many brain regions, however, there were no statistically-significant changes in any ROI (Supplementary Table 4) perhaps, again, due to high inter-individual variability (Supplementary Table 3).

Collectively, from these data it was clear that averaging fluorescence intensity across the whole experimental period resulted in the loss of resolution and statistical power, with respect to relative regional changes in neural activation or suppression following drug treatment. Consequently, the detailed analysis of resultant waveforms within each region was required in order to reveal the more subtle effects of treatment with these 4 pharmacologically-distinct chemoconvulsant drugs.

### Mapping whole brain neural activity during drug treatment - temporal profile waveform analysis

**Figure 5:**
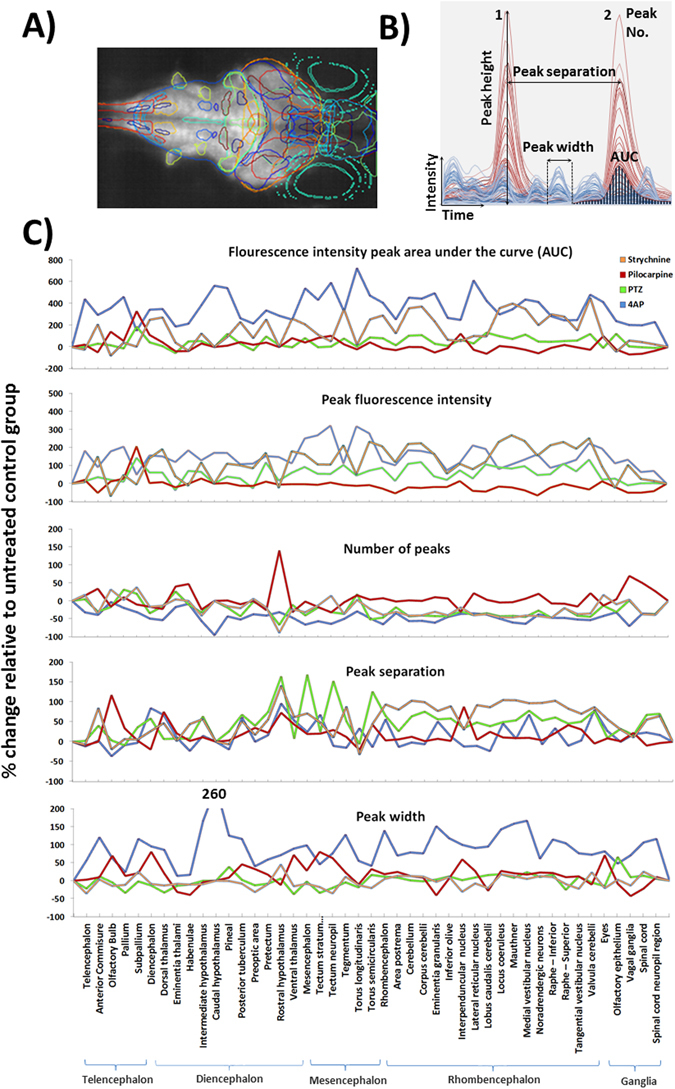
Results of the profile peak analysis. (**A**) Maximum intensity projection through a larva with overlays showing the positions of the 45 registered anatomical regions. (**B**) The various peak analysis parameters automatically quantified for each registered anatomical region, within each fish, are defined in panel B. For illustrative purposes only partial example profiles for a PTZ-exposed (red) and control fish (blue) are shown and the various parameters highlighted on these traces. (**C**) Peak profile analysis data for each region across treatments. The treatment group shown is that at which the highest activity was observed (Top concentrations for 4AP, pilocarpine and strychnine, and 2.5 mM for PTZ due to a slight dip in neural activity at the highest treatment level) Each data point is the mean across all fish/treatment group expressed as the % change versus the mean of the corresponding control group (F_1_ − F_0_)/F_0_*100, where F_1_ = treated group value, and F_0_ = control group value. Note that for the AUC and the peak height profiles, 4AP data were divided by 10 to allow plotting on the same axis. Data also summarised in Supplementary Figure 5.

A summary of the data obtained from the temporal waveform profile analysis is provided within Supplementary Figure 5, and graphically presented in Fig. 5.

**Figure 6:**
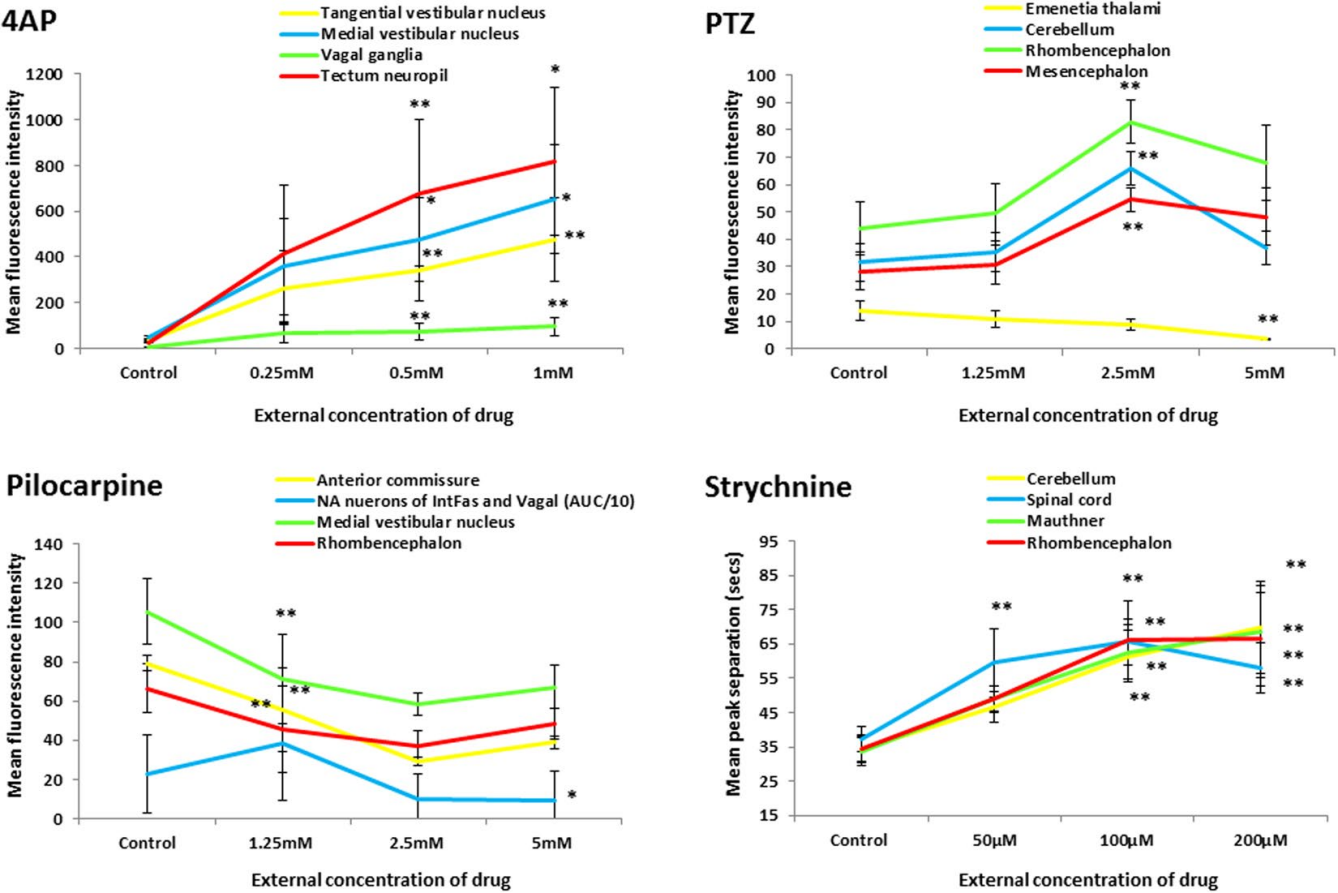
Example concentration response curves generated for selected brain regions of interest in fish exposed to each of the 4 drugs. To provide representative curves, all data shown are those derived from measurements of mean peak fluorescence intensity across all larvae within that treatment group, except pilocarpine noradrendergic (NA) neurons of the interfascicular and vagal areas for which the AUC is presented (divided by 10 to allow plotting on the same axis) and all of the strychnine data (peak separation data shown). Data are shown as the average of the median fluorescence intensity measures obtained for each fish in that treatment group, ±SEM (n = 7–8). All statistical analyses were undertaken using a Kruskal Wallis analysis across treatment groups, followed by a Dunn’s post-hoc test in which each drug-treated group was compared with the corresponding control group. *Denotes significance at the p < 0.05, and ** at the P < 0.01 level in order of lines on graph. Strychnine significance levels are shown below each point in order of the legend. For full concentration-response datasets please see Supplementary Table 5.

Compared with untreated larvae, those exposed to 4AP exhibited an overall trend for highly increased AUC, peak heights (Fig. 5, note this is divided by 10 to allow plotting on the same scale) and peak width. Corresponding with this was a widespread reduction in peak number and increased separation suggesting that 4AP-exposed animals showed larger and more intense (area, duration and intensity), but less frequent, bursts of neural activity in multiple brain regions compared with those in control animals. These trends were further supported by a number of statistically-significant, concentration-related changes within specific brain regions (Supplementary Table 5). Significant concentration-related increases in AUC and peak height were observed most consistently in mesencephalic and rhombencephalic regions, and were especially centred on tectal, cerebellar and vestibular centres as well as within the vagal ganglia (example concentration response curves shown in Fig. 6). Similar significant changes were observed with respect to peak width and number further supporting the induction of fewer, larger spikes of activity occurring in a concentration-dependent manner (although no significant effects in peak separation were detected in any brain region assessed, see Supplementary Table 5).

Larvae exposed to PTZ (Fig. 5) generally exhibited increases in AUC and peak height, together with an increase in peak separation and decreased peak number (note peak activity was at 2.5 mM rather than 5 mM). This again suggests less frequent bursts of higher intensity activity in particular regions compared with untreated larvae. The areas exhibiting the most consistently significant elevations in peak intensity and AUC (Supplementary Table 5) were centred on the rhombencephalon, particularly in sub-regions of the cerebellum, in addition to some regions of the mesencephalon (example concentration-response curves shown in Fig. 6). However, few significant changes in peak width were observed. Conspicuously, only 2 regions exhibited significant reductions in activity (the preoptic area and the emenentia thalami), both of which were in the diencephalon (Fig. 6 and Supplementary Table 5). As stated, increased intensity was mirrored with significant reductions in peak number and increased peak separation across many of the same areas (Supplementary Table 5).

Following exposure to pilocarpine, larvae generally exhibited decreased peak height and AUC, along with decreased peak number and increased separation, but with a concomitant increase in peak width (Fig. 5). This overall pattern suggested a reduction in event intensity, magnitude and frequency in pilocarpine-treated larvae, alongside a slight increase in event duration. These changes were observed predominantly in the rhombencephalon, but also in the tegmentum, spinal cord neuropil region and the anterior commissure. These trends were supported by statistically-significant responses in a number of defined brain regions (Supplementary Table 5 with example curves in Fig. 6). In most cases, however, these effects were significant only at the lower or intermediate treatment concentrations.

Larvae exposed to strychnine exhibited a general trend for elevated AUC and peak height (Fig. 5 and Supplementary Figure 5), however, none of the ROIs exhibited a statistically-significant change in activity when compared with the corresponding untreated control larva data (Supplementary Table 5). In addition, there was a concomitant trend for reduced peak number and increased separation and variable changes in peak width in strychnine-treated animals. A number of ROIs exhibited a significant concentration-related decrease in event number and increase in separation (Supplementary Table 5) compared with untreated larvae. Many of the concentration-related changes in peak number and separation were confined to sub-regions of the rhombencephalon and spinal cord whereas areas showing a significant reduction in peak width were conspicuous by the dominance of the mesencephalon (example curves shown in Fig. 6).

### *In vivo* electrophysiology

**Figure 7:**
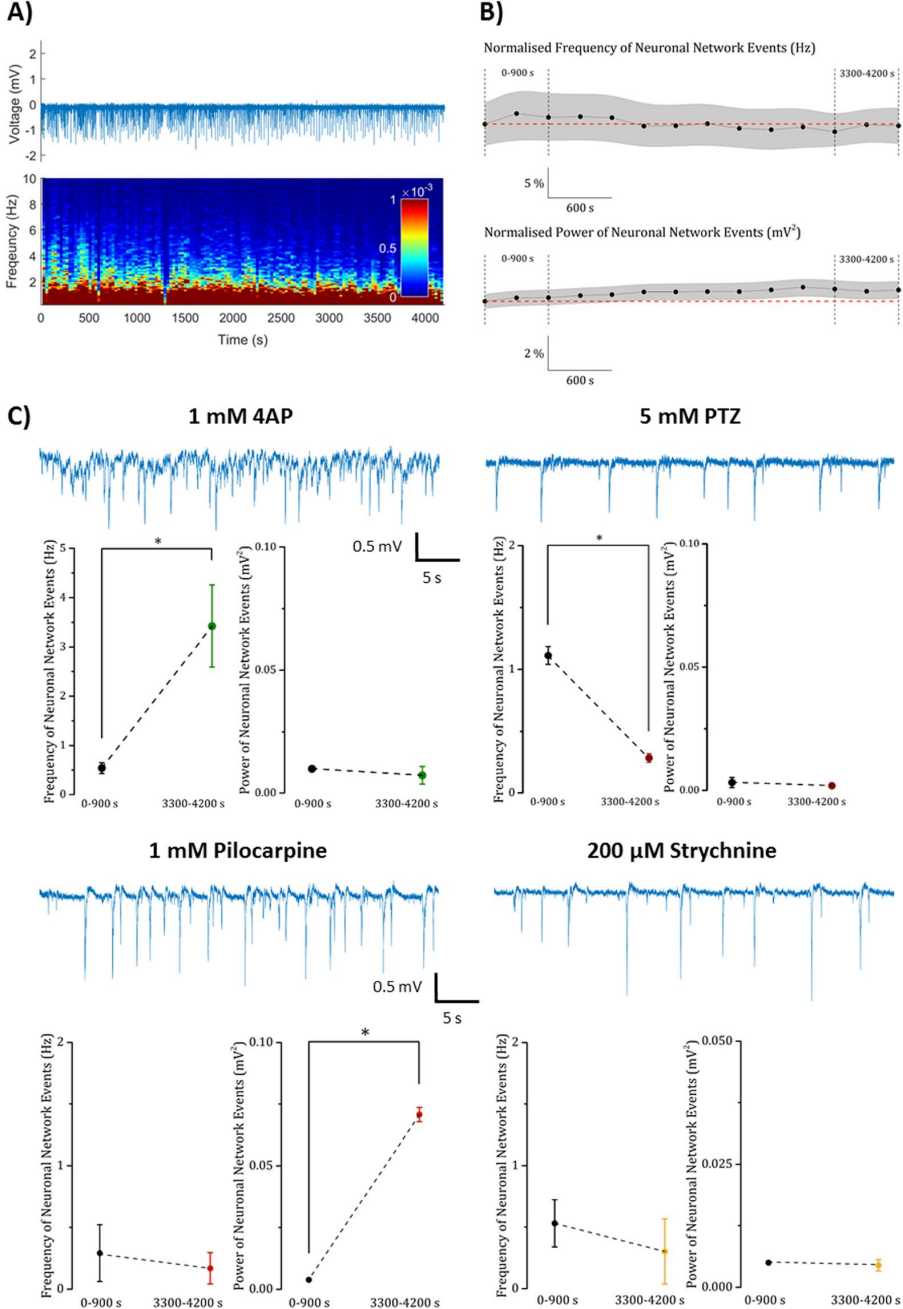
Summary of the electrophysiology data obtained after exposure to the 4 drugs. Panel (A) Experimental trace and supporting spectrogram of basal waveform activity recorded from the optic tectum of untreated control zebrafish larvae. (**B**) The baseline normalised frequency and power of neuronal network events in untreated control larvae does not change over the time course of the experiment (n = 6 zebrafish). (**C**) Representative traces obtained from larvae exposed to the 4 exemplar neuroactive drugs and below each trace, graphs showing control versus treated fish neural event frequency and power. In each case, data are shown as the Mean ± SEM (n = 3 larvae, except pilocarpine = 2). Baseline and post-treatment traces were compared using paired Student’s t-test from which * denotes a significant difference at the P < 0.05 level.

A summary of the data obtained from electrophysiological recordings in drug-treated 4dpf *elavl3*:*GCaMP6s* larvae are presented in Fig. 7, with full results contained within Supplementary Figures 6–10. Following application of 1 mM 4AP, the frequency of neuronal network activity was significantly increased (P < 0.05), however there was no significant effect on power. Interestingly, the significant increase in tectal region AUCs observed in our functional imaging may reflect this elevated spiking across large neuronal populations resulting in large scale GCaMP fluorescence increases, with little time for recovery in between. Exposure to 5 mM PTZ resulted in reduced event frequency (P < 0.05) with no effect on power. The former mirrored the observed reduction in tectal fluorescence intensity, peak number and frequency detailed above. In contrast, pilocarpine exposure (1 mM) resulted in an increase in event power (P < 0.05), but not in frequency, and exposure to 200 μM strychnine resulted in no significant changes in either parameter. The imaging data for pilocarpine suggested relatively widespread reduction in activity, however this was predominantly observed in the rhombencephalon. Similarly, the absence of any effect of strychnine exposure perhaps reflects the relatively low occurrence of GlyR in mid- and forebrain regions (see Discussion), and both of these points illustrate the issue of relatively low spatial coverage when using electrophysiological recording approaches to gather information on the large-scale modulation of neural networks.

## Discussion

### The power of transgenic zebrafish and LSM for functional 4D drug profiling in the vertebrate brain

Here we show that the combined use of a pan-neuronal, genetically-encoded Ca^2+^ sensor in transparent larval zebrafish with LSM affords an extremely powerful approach for the quantitative functional analysis of vertebrate neural networks, following drug treatment.

A key limiting factor in interpreting LSM-generated neural function data has been the efficient transformation and 3D anatomical registration of the many resultant images. This has posed a significant challenge for accurately identifying ROIs and associated activity profiles when quantifying treatment-related effects^4^. We have overcome this by developing and applying a novel Python image processing pipeline in which we are able to automatically superimpose captured image voxel data onto a standardised 3D anatomical map of the larval zebrafish brain^23^ and extract spatiotemporal profile data for each registered brain region. Our methodology allows us to quantify treatment-related changes in neural function within and between specific ROIs, and to compare neuropharmacological profiles between different treatment regimes. We have demonstrated, using selection of chemoconvulsant drugs, that this type of approach is suitable for the identification and quantification of activity across large-scale, integrated neuronal assemblies, and greatly expedites the time and region-directed analysis of network activation after drug treatment.

### Control animal activity is associated with arousal, alertness, and escape responsiveness

In untreated control larvae, there was a high degree of consistency in brain regions showing low level elevation of GCaMP fluorescence, compared with the corresponding baseline intensity. Most of these regions were within the rhombencephalon, most prominently the inferior olive, locus coeruleus, Mauthner cells, raphe and medial vestibular nucleus. Consistent with the nature of our conscious, but immobilised, preparation, these regions are notable for their functional roles in sensory processing, arousal, and the escape response^24, 26–30, 35^. Some this of this low level activity observed could indeed reflect network-wide responses to accelerational, photic and or auditory stimulation that may be present due to movement of the stage and/or laser illumination during imaging. Further supporting this, the tegmentum was the only mesencephalic region showing consistently high activity in control larvae, having a key role in the coordination of sensory inputs and motor control^33, 34^, and particularly high activity was also noted in the anterior commissure in the telencephalon, and the caudal hypothalamus in the diencephalon which are involved in functional connectivity^31^ and autonomic process control^32^ respectively. The inferior olive, which is thought to be essential for adaptive motor control^24^, also showed consistent above-baseline levels of spontaneous neural activity in control animals. Interestingly, a number of previous authors^9, 10, 36^ have reported the existence of a ‘hindbrain oscillator’ adjacent to the inferior olive that exhibited ‘slow lateralized oscillations’ in larvae in the absence of any exogenous sensory stimulation. In support, the coefficient of variation of peak frequency in the inferior olive, across all control larvae, was 33% (n = 32), suggesting a relatively consistent temporal activity profile in this region. Furthermore, the mean frequency, in seconds (38, n = 32) is broadly in line with the oscillatory period of 20–30 s reported by Ahrens *et al*.^9^. Collectively these data support the suitability of our approach with respect to the identification and quantification of neural signals within discrete brain structures.

### Different pharmacological mechanisms elicit distinct spatiotemporal neural activity patterns

Exposure to the K^+^ channel blocker 4AP resulted in considerable and widespread GCaMP activation, particularly in tectal and cerebellar nuclei. Very little is documented regarding the distribution of Kv1 channels and associated neural circuitry, or the activity of K^+^-blockers in the zebrafish CNS, however, Fosque *et al*.^37^ recently published a confocal image of CNS activity following exposure to 800 μM 4AP, from transgenic zebrafish larvae expressing the novel photoconvertible Ca^2+^ sensor CaMPARI. CaMPARI expression was evident throughout the brain and in particular the hindbrain and spinal cord following 4AP treatment, although, due to the nature of the CaMPARI reporter, the temporal profile and regional quantification of activity were not available for close comparison with our data. In rats injected with 4AP or picrotoxin (a PTZ-like GABA_A_ antagonist), Salami *et al*.^38^ reported distinct EEG patterns: GABA_A_ antagonism resulted in rapid focal onset and dissipation, whereas 4AP administration resulted in more diffuse activity. Furthermore, it was noted that high frequency oscillations differed between 4AP and picrotoxin: fast ripples were characteristic of picrotoxin exposure (thought to reflect hypersynchronous firing of principle glutamatergic neurons); and slow ripples characteristic of 4AP exposure. These descriptions resonate with the evolution of network hyperactivity seen here (see also Supplementary Videos 2 and 4), in which 4AP treatment resulted in a relatively slow progression of GCaMP activation across the entire brain, whereas PTZ treatment resulted in sharp rhythmic spiking within discrete brain regions from the onset. Distinct *in vivo* electrophysiological profiles were also obtained following 4AP and PTZ treatment: PTZ-induced network activity was regular, consistent and discrete, whereas 4AP-induced network activity was more frequent and variable in terms of magnitude. When considering these profiles alongside the functional imaging output, in most cases, the 4AP imaging data revealed a small number of large magnitude increases in GCaMP fluorescence across multiple brain regions (see also Supplementary Video 2) perhaps suggesting that the temporal resolution of the imaging was insufficient to capture the faster temporal components seen with *in vivo* electrophysiology; rather it manifested as a single global elevation in GCaMP fluorescence. This possibility highlights the relative advantages and disadvantages of each approach, namely the superior spatial resolution juxtaposed with inferior temporal resolution of the functional imaging versus *in vivo* electrophysiology. It is also important to reiterate that, although both methods report neural activity, electrical recordings and GCaMP-based imaging do not measure identical phenomena. Pivotally, extracellular electrical recording can effectively report sub-threshold electrical activities, for example those arising from synaptic activity, and in particular synchronised synaptic activity; whereas GCaMP signals will predominantly arise from suprathreshold membrane potential changes and the voltage-gated Ca^2+^ entry they drive. Also the influence of the kinetics of Ca^2+^ binding and particularly unbinding to/from GCaMP determine the nature of how it can report neural activity.

GABA-immunoreactive cells are found in the CNS of zebrafish early in development, and by 3dpf their distribution is similar to that of comparatively developed mammals^39, 40^. The reported distribution shows some similarities with our temporal analysis-derived peak height and AUC data, which suggest removal of inhibitory synaptic drive triggering neural hyperactivity in a number of prominent regions. In support, Mueller *et al*.^40^ reported many GABA-immunoreactive (-IR) cells in the olfactory bulb and subpallium, with some penetration of the pallium; all regions showing elevated GCaMP activity here following PTZ-treatment. These authors also reported GABA-IR cells across the diencephalon, but noted relative absences in the anterior preoptic area and emenetia thalami, both areas of which showed conspicuous reductions in peak GCaMP fluorescence after PTZ treatment. These authors also reported detailed thalamic expression of GABA-IR: higher levels were observed in posterior parts of the dorsal and ventral thalamus into the prectectum (which is the only one of these regions showing a significant increase in peak height in our data), compared with a relative absence in the anterior portions of these regions. Although we are able to provide region-specific fluorescence intensity measures, our registration method does not differentiate between posterior and anterior sections of such regions. At present we can only quantify median intensity across the whole region which may mask sub-regional variations in expression revealed using GABA-IR. Rhombencephalic regions reported as having notable GABA-IR cell populations by Mueller *et al*.^40^ centred on the cerebellum and sub regions, such as the valvula and corpus cerebelli. Our data also showed elevated GCaMP fluorescence in these regions which was consistently higher than control levels, with respect to both peak height and AUC from the temporal profile peak analysis. A key consideration to bear in mind when comparing the output from our functional imaging approach, versus descriptions of the distribution of neurotransmitters or their target receptors, is that our approach provides quantitative data on whole network activation and suppression. For example, each neural target possesses a network of stimulatory or inhibitory inter-neuronal connections with other regions, which may be functionally activated regardless of the presence or absence of target receptors. Similarly each of these has multiple downstream regional activation profiles, which may be targeted by the neural circuit under investigation, as well as other circuits and inputs independent of the initial target network. Consequently, the presence or absence of immunoreactivity in a specific region is not necessarily a predictor of activation after pharmacological treatment. As such our method’s key strength is in providing a greater understanding of network structure and regional cross-talk than is achievable using non-functional imaging-based approaches. This allows us to understand which neural circuits are affected by drug treatment, rather than which act as the initiator or initial propagator of such activity.

No comparable zebrafish functional imaging data are available. However, Van Camp *et al*.^41^ employed functional Magnetic Resonance Imaging (MRI) in rats dosed with PTZ and reported enhanced activity in cortical regions, the hypothalamus and brain stem. They also identified that the neocortex (analogous to the dorsal pallium in zebrafish) was strongly involved in PTZ-induced hyperactivation. Although we observed some enhanced activity in the telencephalon, there was a relative absence of significant-concentration-related effects on measures of GCaMP fluorescence intensity. One exception was the olfactory bulbs, which exhibited a highly significant elevation in peak height, but only at the lowest PTZ concentration. It could be postulated that this reflects an initial olfactory area response, subsequently superseded by global network hyperactivity at higher PTZ concentrations. As stated, Van Camp *et al*.^41^ also reported elevated MRI signals in the hypothalamus, thalamic regions and ‘brain stem’ although the specific regions were not revealed. Here, (hypo)thalamic regions by and large exhibited modest, non-significant changes, but a number of mid- and hindbrain structures showed significant concentration-related increases in GCaMP fluorescence after exposure to PTZ, particularly focussed on the tectum and cerebellum.

Pilocarpine exposure typically resulted in a trend for reduced GCaMP fluorescence, particularly in the rhombencephalon. No comparable zebrafish functional imaging data are available and data on the occurrence and distribution of mAChRs in zebrafish are limited. Park *et al*.^42^, however, showed that the mixed mAChR/nicotinic receptor agonist carbachol reversibly suppressed electrically-evoked field potentials in the isolated telencephalon of adult zebrafish in a dose-dependent manner. These authors noted that, in the rat telencephalon, mAChR activation has opposing effects due to its inhibition of K^+^ conductance resulting in neuronal excitation, and simultaneous activation of GABAergic inhibition resulting in neuronal depression. Furthermore, this effect is carbachol-concentration dependent in rats, with enhancement of evoked activity predominant at low concentrations and the opposite true at higher concentrations. It is possible, therefore, that our observations of reductions in GCaMP fluorescence across multiple regions reflect inhibitory dominance due to the relatively high concentrations of pilocarpine used. In contrast with the predominant inhibition of GCaMP fluorescence across mesencephalic and rhombencephalic regions, some forebrain regions exhibited a trend for modest (non-significant) elevations in GCaMP fluorescence. Interestingly, the *in vivo* electrophysiology exhibited a significant increase in event power in the optic tectum following pilocarpine treatment. Although perhaps surprising given the modest elevation in GCaMP activity observed in the forebrain, as discussed previously, the two methods do not provide identical readouts. In addition, electrophysiological recordings sample small sub-populations of cells within the wider region, whilst GCaMP fluorescence data reflect the median intensity value for that whole region within which isolated populations of cells may show much higher localised activity in response to drug treatment (for example as observed in the subpallium). This further highlights the difficulty in interpreting electrophysiological data, with its low spatial resolution and coverage, in the context of whole, or even regional, brain functionality.

Strychnine exposure resulted in a general trend for elevated time-averaged GCaMP activity. However, despite the apparent widespread activation across multiple regions, the responses exhibited between animals were variable, and no significant effects on peak height or AUC were observed. Assessment of the significant effects of strychnine treatment on other peak parameters revealed that, in the vast majority of cases, these were confined to the hindbrain and spinal cord. This pattern is consistent with the role of glycine as the major inhibitory neurotransmitter of the caudal CNS, reflected in the distribution of the GlyR which shows a clear rostral to caudal increase in expression in both mammals^43^ and zebrafish^44, 45^. Despite this, however, there were still no significant increases in GCaMP activity (median intensity, AUC or peak height) within hindbrain and spinal cord regions, perhaps due to the relatively high variability in responsiveness shown between individual animals. A lack of a significant effect, on either event power or frequency, was also evident from the *in vivo* electrophysiology following strychnine treatment. This however, is more easily explained due to the likely low density of GlyR in the optic tectum of these animals, and the predominant role of glycine in hindbrain and spinal cord neurotransmission. Interestingly, Malosio *et al*.^46^ observed that rat GlyR expression was abundant amongst cell populations known also to express GABA_A_ receptors, and speculated that coexpression may allow more precise control of inhibitory inputs. Our findings may also support this at a functional network level. When considering the time-averaged median intensity data, the spatial distribution of regions showing altered neural activity after exposure to both PTZ and strychnine were broadly similar, but with the generally lower activity evident in strychnine-exposed larvae perhaps reflecting the lower density of GlyR compared with GABA_A_ receptors in midbrain structures.

Two regions that were consistently (significantly) affected across all drug-treated groups were the Mauthner cells and the medial vestibular nucleus. Mauthner cells in the Goldfish have been shown to be sensitive to the effects of strychnine, the GABA_A_ antagonist bicuculline, as well as the voltage-gated potassium channel blocker dendrotoxin I^47^. This is of interest as exposure here, to representatives of both of these pharmacological classes, resulted in evidence of elevated GCaMP fluorescence and thus neural activity in the same discrete region. The fact that both the Mauthner cells and vestibular nuclei appear to be consistently affected (either positively or negatively) is perhaps unsurprising given the nature of our conscious larval preparation, and that multiple auditory and vestibular afferents are known to innervate Mauthner cells in larval zebrafish^48^.

### The future of functional neuropharmacological profiling in zebrafish

Despite the small scale of the zebrafish brain, there is still currently a trade-off between spatial and temporal resolution for applications focussed on whole brain functional profiling^49^. Our approach was appropriate for the capture of neural activity within specific brain regions following treatment with our selected drugs, however, faster acquisition rates would allow greater temporal resolution as well as higher spatial resolution at any given acquisition rate. In this respect, the use of piezoelectric drives to move illumination and detection objectives relative to a static sample holder to minimise accelerational forces, in combination with a faster camera, would allow more rapid cycle times. Another consideration is the differential expression of GCaMP across different anatomical regions. Arrenberg and Driever^4^ pointed out that the *HuC/elavl3* promoter induces relatively low levels of expression in the diencephalon at 5dpf, and that GCaMP expression is cytosolic rather than nuclear. Although the use of untreated versus treated groups, and baseline-corrected control animal data allowed comparison of relative activity levels within regions here, diencephalic regions do appear underrepresented in terms of significant concentration-related effects, especially when assessing the peak analysis data. Analysis of the % fluorescence intensity (% of the average across the whole brain) exhibited by each broad brain region compared with the overall average for all regions, reveals that the diencephalon, mesencephalon and rhombencephalon exhibit 75%, 111% and 127% of the whole brain mean fluorescence respectively, whereas the telencephalon and ganglia exhibit just 39% and 52% respectively (Supplementary Table 6). It is not possible to say whether these regional differences reflect low inherent expression or low activity. The occurrence of significantly elevated activity within specific structures from all of these broad regions following drug treatment, however, suggests that any differential expression did not hamper our ability to detect the effects of the drugs that were applied. Nevertheless, such approaches would benefit greatly from the identification of other promotors^4^, particularly those allowing fluorophore-based differentiation between brain regions. The cytosolic expression of GCaMP6s also has the potential to confound precise cellular location of neural activity^4^. For our neuropharmacological profiling approach, which focusses on broad scale expression patterns and the analysis of dynamic neural network activation and suppression, this factor is less important. However, single cell, and even subcellular resolution is clearly achievable using multi-photon based systems (see Supplementary Figure 11 and Video 5), and therefore for experimental paradigms that require cellular detailed assessment, nuclear and other compartmentalised sensors would be desirable.

In summary we have demonstrated the power of using a pan-neuronal genetically-encoded Ca^2+^ sensor in transparent larval zebrafish, along with LSM, for profiling integrated system-wide neural circuit responses to treatment with CNS-active drugs in a highly relevant vertebrate model. Our approach has clear potential for application to many areas of CNS-focussed research, particularly when combined with emerging technologies that have greatly simplified *in vivo* gene editing in the zebrafish^50^. In the current context of compounds resulting in neural network hyperexcitability, this includes: the identification of convulsive properties and their respective mechanisms as side effects of new drugs, by allowing comparison of resultant neural activity patterns against a library of known pharmacology-linked phenotypes; for testing the efficacy and identifying novel mechanisms of action of anticonvulsive drugs; and for aiding in the clarification of poorly understood molecular mechanisms. More generally this approach has considerable potential in research encompassing the assessment of spatiotemporal neural network functionality in the normal and diseased vertebrate brain.

## Electronic supplementary material

Supplementary information

Supplementary Video 1

Supplementary Video 2

Supplementary Video 3

Supplementary Video 4

Supplementary Video 5
