# Author Correction: 4-dimensional functional profiling in the convulsant-treated larval zebrafish brain

**DOI:** 10.1038/s41598-018-32731-5

**Published:** 2018-10-23

**Authors:** Matthew J. Winter, Dylan Windell, Jeremy Metz, Peter Matthews, Joe Pinion, Jonathan T. Brown, Malcolm J. Hetheridge, Jonathan S. Ball, Stewart F. Owen, Will S. Redfern, Julian Moger, Andrew D. Randall, Charles R. Tyler

**Affiliations:** 1 Biosciences, College of Life and Environmental Sciences, Exeter, Devon, EX4 4QD United Kingdom; 2 Medical School, University of Exeter, Exeter, Devon, EX4 4PS United Kingdom; 3 AstraZeneca, Global Compliance, Alderley Park, Macclesfield, Cheshire, SK10 4TF United Kingdom; 4 AstraZeneca R&D Innovative Medicines, Drug Safety & Metabolism, Babraham Research Campus, Cambridge, CB22 3AT United Kingdom; 5 Physics and Medical Imaging, College of Engineering, Mathematics and Physical Sciences, University of Exeter, Exeter, Devon, EX4 4QL United Kingdom

Correction to: *Scientific Reports* 10.1038/s41598-017-06646-6, published online 26 July 2017

This Article and the accompanying Supplementary information file contain a repeated labelling error where,

‘anterior commissure’

was mislabelled and should read:

‘Spinal cord (Vmat2 Stripe1 region)’.

As a result, the correct Figure 2 and its accompanying legend appear below.

**Figure 2:**
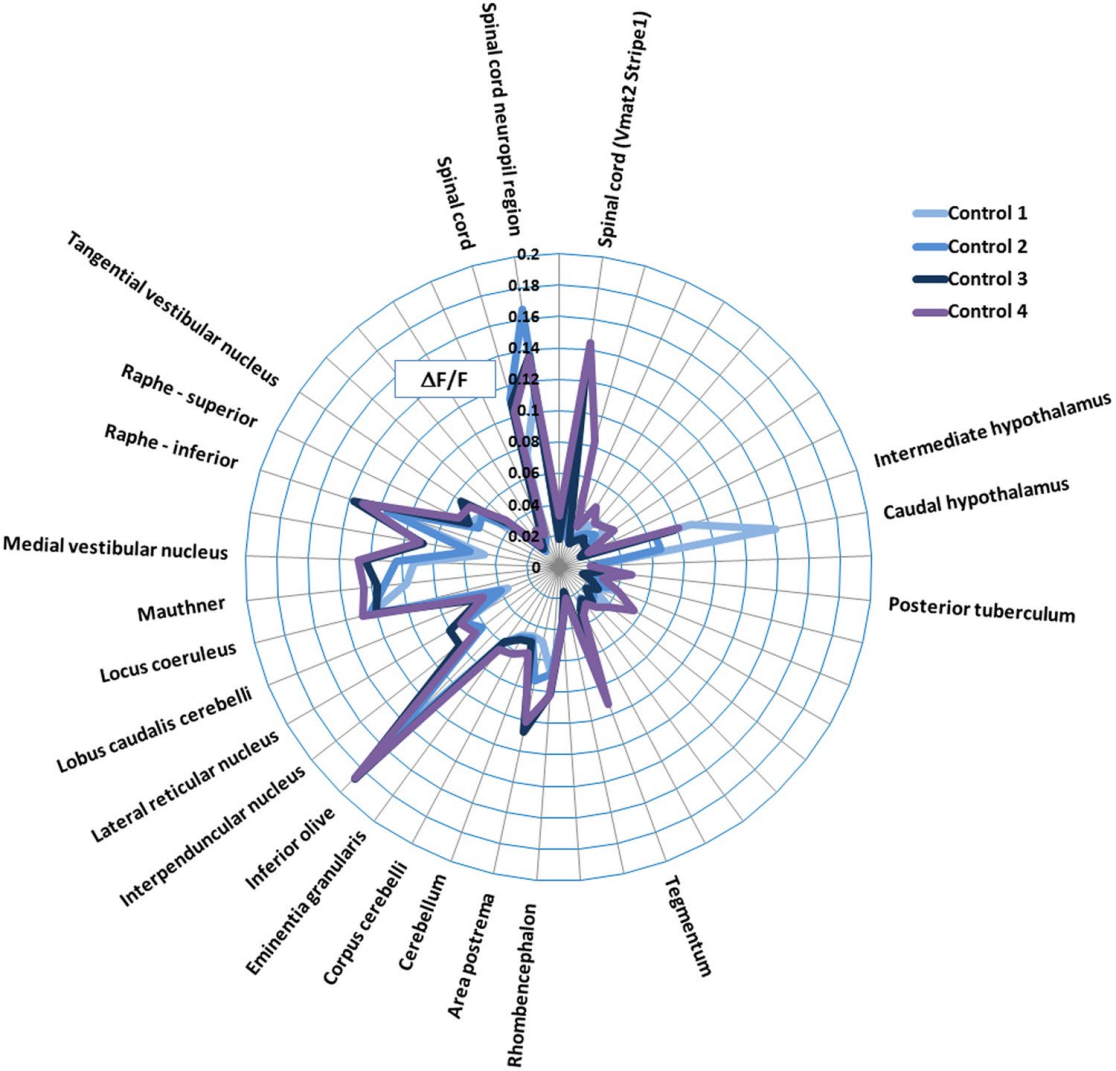
Summary of the control fish data across 4 experiments. Data are shown as the average of DF/F (=(F_1_−F_0_)/F_0_*100, where F_1_ = peak fluorescence intensity, and F_0_ = baseline fluorescence intensity) of the fish within each control group. Only those regions showing activity greater than the median of all areas (>0.0386) are labelled. Note the consistency in brain regions showing measurable baseline activity. In particular, activity was most pronounced in the hypothalamus, tegmentum, rhombencephalon, inferior olive, locus coeruleus, mauthner cells, medial vestibular nucleus, superior and inferior raphe, spinal cord neuropil region and spinal cord Vmat2 Stripe1.

The correct Figure 4 also appears below.

**Figure 4:**
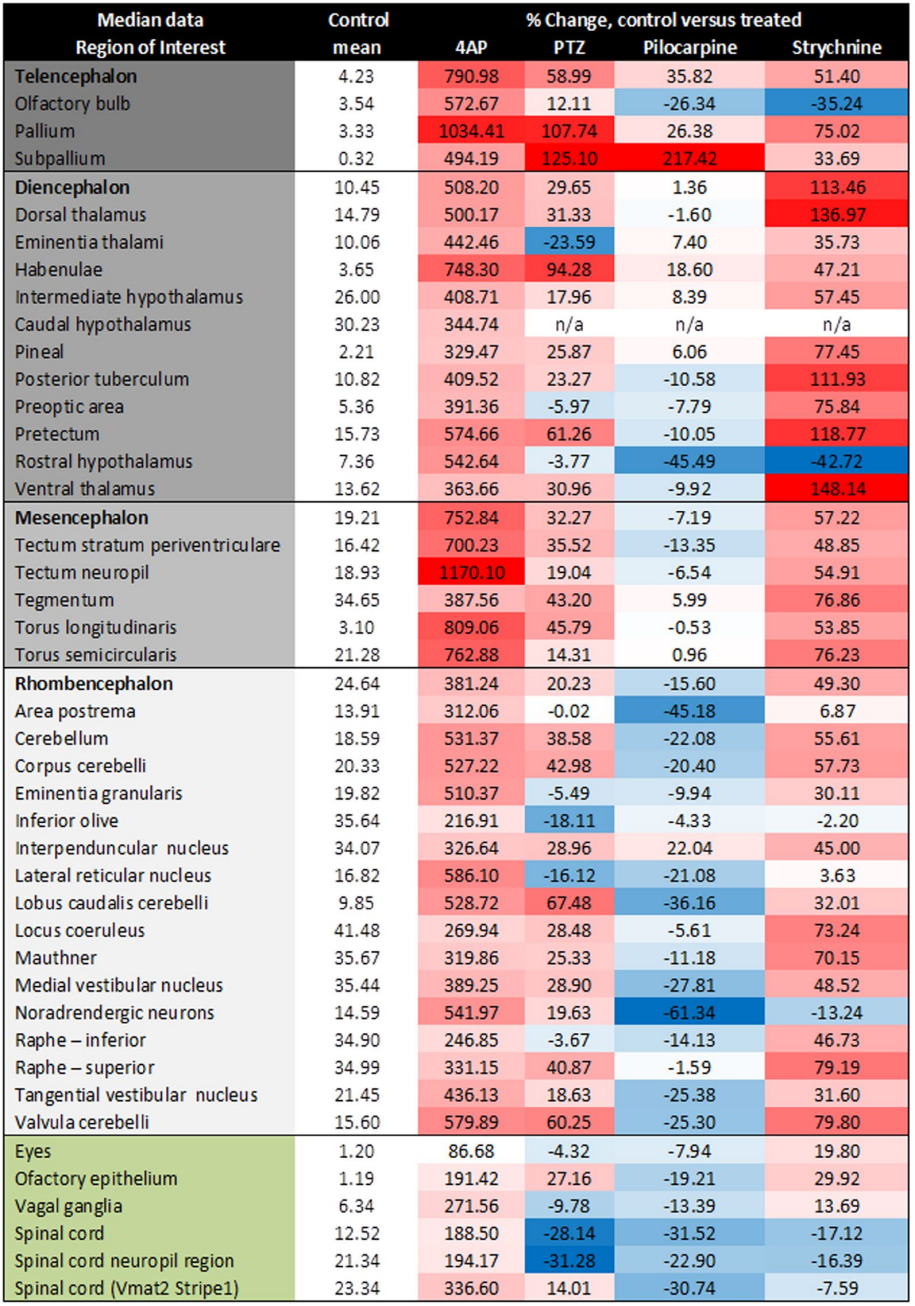
Summary of the voxel GCaMP6s fluorescence intensity obtained per anatomical region for each of the model chemoconvulsant compounds applied. Data are expressed as the time-averaged median fluorescence intensity values, averaged across all fish in that treatment group, expressed the % change versus the corresponding control fish group (second left column presented for brevity as the mean across the 4 control groups): ΔF/F = (F1−F0)/F0*100 (where F1=treated group fluorescence intensity, F0=control group fluorescence intensity). Colour coding represents the degree of activation (shades of red), or suppression (shades of blue) versus that region in the control larvae, relative to other brain regions within that treatment group. The treatment group shown is that at which the highest activity was observed, which was the top concentration for 4AP, pilocarpine and strychnine, but 2.5 mM (the second highest) for PTZ due to a slight dip in neural activity at the highest treatment level. Colour coding in the left most column represents anatomical categorisation as subdivisions of the telencephalon, diencephalon, mesencephalon and rhombencephalon, along with ganglia (green) respectively from top to bottom. n/a – values not obtained due to a failure in registration probably due to the high z-depth of this region. N = 8 larvae were imaged per group. For corresponding SEM values, see Supplementary Table 3.

The correct Figure 5 appears below.

**Figure 5:**
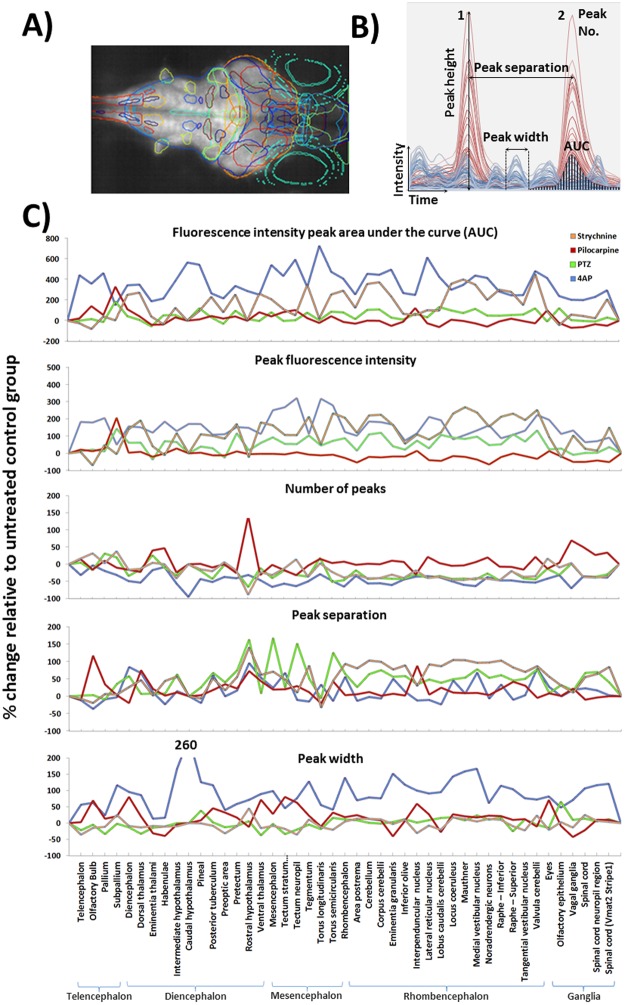
Results of the profile peak analysis. (**A**) Maximum intensity projection through a larva with overlays showing the positions of the 45 registered anatomical regions. (**B**) The various peak analysis parameters automatically quantified for each registered anatomical region, within each fish, are defined in panel B. For illustrative purposes only partial example profiles for a PTZ-exposed (red) and control fish (blue) are shown and the various parameters highlighted on these traces. (**C**) Peak profile analysis data for each region across treatments. The treatment group shown is that at which the highest activity was observed (Top concentrations for 4AP, pilocarpine and strychnine, and 2.5 mM for PTZ due to a slight dip in neural activity at the highest treatment level) Each data point is the mean across all fish/treatment group expressed as the % change versus the mean of the corresponding control group (F1−F0)/F0*100, where F1=treated group value, and F0=control group value. Note that for the AUC and the peak height profiles, 4AP data were divided by 10 to allow plotting on the same axis. Data also summarised in Supplementary Figure 5.

Finally, the correct Figure 6 appears below.

**Figure 6:**
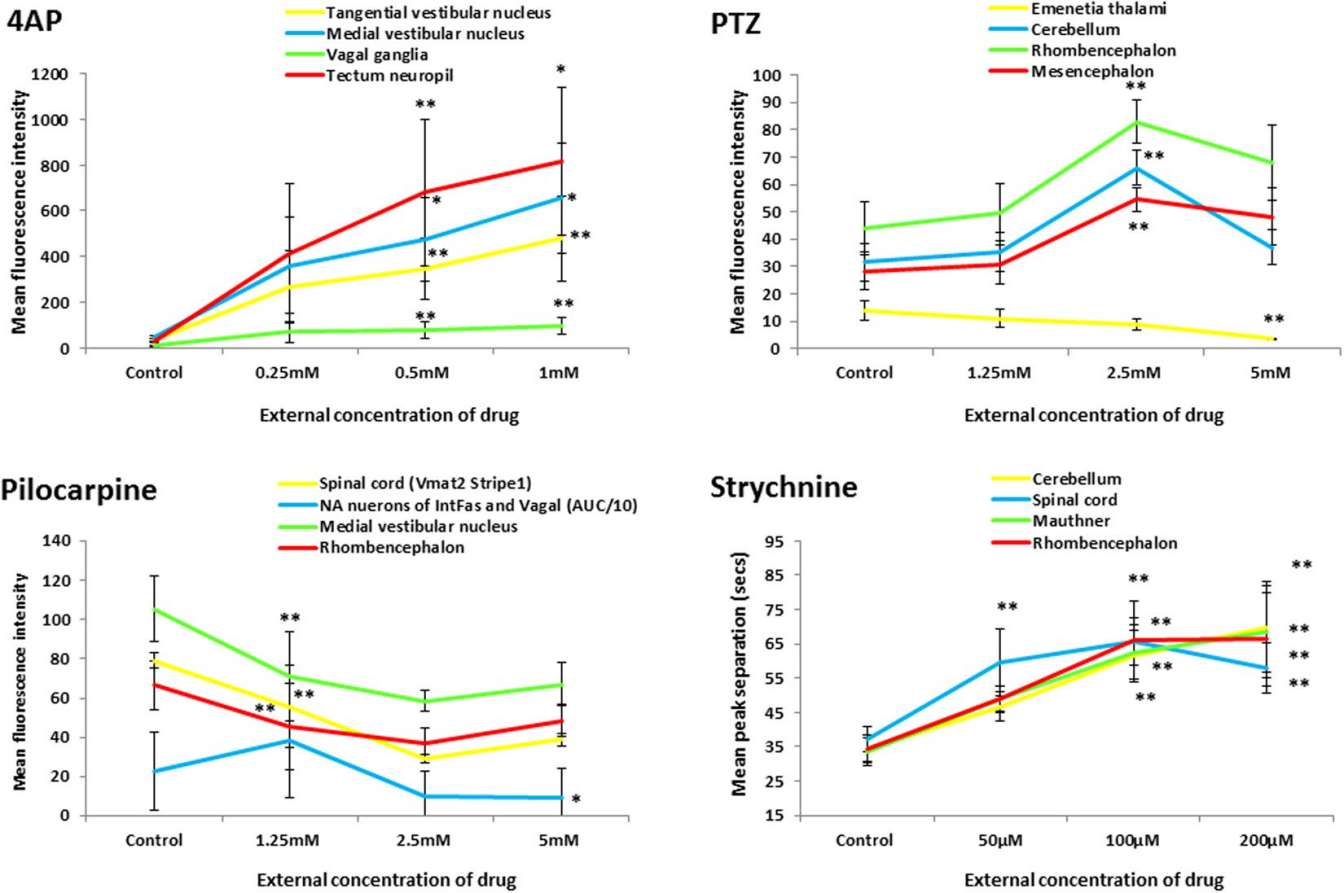
Example concentration response curves generated for selected brain regions of interest in fish exposed to each of the 4 drugs. To provide representative curves, all data shown are those derived from measurements of mean peak fluorescence intensity across all larvae within that treatment group, except pilocarpine noradrendergic (NA) neurons of the interfascicular and vagal areas for which the AUC is presented (divided by 10 to allow plotting on the same axis) and all of the strychnine data (peak separation data shown). Data are shown as the average of the median fluorescence intensity measures obtained for each fish in that treatment group, ±SEM (n = 7–8). All statistical analyses were undertaken using a Kruskal Wallis analysis across treatment groups, followed by a Dunn’s post-hoc test in which each drug-treated group was compared with the corresponding control group. *Denotes significance at the P < 0.05, and **at the P < 0.01 level in order of lines on graph. Strychnine significance levels are shown below each point in order of the legend. For full concentration-response datasets please see Supplementary Table 5.

