# Supplementary material for: 4-dimensional functional profiling in the convulsant-treated larval zebrafish brain
Source: Sci Rep. 2017 Jul 26;7:6581. doi: 10.1038/s41598-017-06646-6 (PMC5529444; doi:10.1038/s41598-017-06646-6)

4-dimensional functional profiling in the convulsant-treated larval zebrafish brain

Authors

Matthew J Winter[1]*, Dylan Windell[1], Jeremy Metz[1], Peter Matthews[2], Joe Pinion[2], Jonathan T. Brown[2], Malcolm J Hetheridge[1], Jonathan S. Ball[1], Stewart F. Owen[3], Will S. Redfern[4], Julian Moger[5], Andrew D. Randall[2], and Charles R. Tyler[1].

Affiliations

[1]Biosciences, College of Life and Environmental Sciences, Exeter, Devon, EX4 4QD, United Kingdom

[2]Medical School, University of Exeter, Exeter, Devon, EX4 4PS, United Kingdom

[3]AstraZeneca, Global Compliance, Alderley Park, Macclesfield, Cheshire, SK10 4TF, United Kingdom

[4]AstraZeneca R&D Innovative Medicines, Drug Safety & Metabolism, Babraham Research Campus, Cambridge CB22 3AT

[5]Physics and Medical Imaging, College of Engineering, Mathematics and Physical Sciences, University of Exeter, Exeter, Devon, EX4 4QL

Supplementary Materials and Methods

Experimental animals

GCaMP6s has a relatively high signal to noise ratio and slower kinetics than other GCaMP forms, exhibiting brighter fluorescence than enhanced-GFP, 10 action potential T_{max} rise and $T_{1/2}$ decay times of 480 and 1796ms respectively (measured in a dissociated neuronal culture), as well as negligible photobleaching over multiple imaging events[1]. Adult *elavl3*:GCaMP6s broodstock were held in flow through aquaria at the University of Exeter, at 28+/- 1°C under optimal conditions for spawning (16 hour light: 8 hour dark cycle, with 20 minute dusk–dawn transition periods). Culture water consisted of mains tap water which was filtered by reverse osmosis (Environmental Water Systems UK Ltd.) and then reconstituted with Analar-grade mineral salts to a standard synthetic freshwater composition (final ion concentrations: 117mg/L $CaCl_2 2H_2 0$, 25.0 mg/L $NaHCO_3$, 50mg/L $MgSO_4 7H_2 0$, 2.3mg/L KCl, 1.25mg/L Tropic Marine Sea Salt, giving a conductivity of 300mS). The culture water was then aerated and heated to 28°+/- 1°C before being supplied to individual zebrafish tanks on a flow through system. This water was routinely monitored for temperature, pH, conductivity, ammonia, nitrite and nitrate, all of which were maintained within suitable conditions for zebrafish. Spawning occurred at first light and was induced through the introduction of a spawning substrate. Fertilized eggs were collected shortly after the spawning event, bleached for 1 minute in 1% w/v chloramine T (Sigma-Aldrich, Poole, UK) in culture water, rinsed with fresh culture water and transferred to Petri dishes which were filled with culture water, and held at 28+/- 1°C, until use in imaging experiments on 4 days post fertilization (DPF).

Prescreening convulsants using wide-field imaging

For these experiments 4 dpf *elavl3*:GCaMP6s individual larvae were immobilized with neuromuscular blocker tubocurarine chloride (TC, Tocris, Bristol, UK) at a concentration of 4 mM, and then mounted dorsal side down in custom designed microchambers in 0.7% low melting point (LMP) agarose (Sigma-Aldrich, Poole, UK). The microchambers containing the immobilized larvae were then transferred to the stage of a standard inverted microscope (Nikon TE2000, Nikon UK Limited, Kingston Upon Thames, UK) which was equipped with a fast switching fluorescent light source (Sutter DG4, Sutter Instrument, Novato, USA) and a sensitive high speed CCD camera (Hamamatsu Orca 12AG, Hamamatsu Photonics UK Ltd, Welwyn Garden City, UK), both under the control of the Volocity imaging software (Perkin Elmer, Seer Green, UK). The light source was used to provide a constant illumination at 490 nm to excite GCaMP6s and the camera was used to collect images of fluorescent output at frame rates of between 5 and 0.2 Hz. Images were typically collected using a 10X fluorescence objective, although a 20X objective was sometimes also employed. Images were collected, and Volocity was used for the analysis of fluorescence intensity versus time, within

defined regions of interest (ROIs). For each subject, typically we collected 4 ROIs, corresponding to the forebrain, the midbrain and the hindbrain, with the fourth ROI selected outside of the subject's body and used for background subtraction purposes. Following recording of a drug-free baseline period of 10 minutes, one of the three test compounds (or vehicle) was added to the microchamber in a volume of 10% of the chamber volume, at a 10x concentration to give a final concentration equal to those shown in **Table 1** in the main manuscript. Following compound addition, the larva was imaged for up to a further 70 minutes. Minimally four 4 dpf larvae were used to assess each compound.

Supplementary Table 1| Maximum tolerated concentration assessment undertaken in 4dpf larvae after one hour of exposure (n=8 larvae per group), in order to determine appropriate (non-lethal) concentrations for further assessment. Higher scores attained in the seeker test are indicative of sedation and motor impairment, abnormal posture is indicative of the loss of balance, unusual morphology includes bending of the tail etc. (see [17] for full methodology). As one can see from these data, the maximum concentrations used in the current study (shaded in grey) were considerably lower than those at which gross toxicity and lethality was observed. Note the impairment of locomotor activity observed after strychnine treatment at multiple concentrations, although the larvae remained alive up to the maximum concentration tested for 1 hour of exposure. Also note the absence of behavioural convulsions exhibited by pilocarpine and strychnine, thus illustrating the value of assessing brain function directly (rather than via behavioural observation) for the detection of drug-induced seizures.

Compound	External concentration	%Mortality	Response to seeker*	%Abnormal posture	%Unusual morphology	% Showing convulsions
4AP	4mM	0	1	12.5	0	87.5
	2mM	0	1	25	0	75
	1mM	0	1.625	25	0	87.5
	0.5mM	0	1.25	0	0	87.5
	0.25mM	0	1.625	0	0	50
	Water	0	1.75	0	0	0
PTZ	20mM	0	1	0	0	75
	10mM	0	1	0	0	100
	5mM	0	1.125	0	0	87.5
	2.5mM	0	1	0	0	100
	1.25mM	0	1.625	0	0	50
	Water	0	1.75	0	0	0
Pilocarpine	40mM	0	2.25	50	0	0
	20mM	0	2	12.5	0	0
	10mM	0	1.25	0	0	0
	5mM	0	1.25	0	0	0
	2.5mM	0	1.125	0	0	0
	Water	0	1.25	0	0	0
Strychnine	800µM	0	3	100	12.5	0
	400µM	0	3	100	12.5	0
	200µM	0	3	100	0	0
	100µM	0	2.875	87.5	0	0
	50µM	0	2	25	12.5	0
	Water	0	1.75	0	0	0

***Scored as follows: 1 = freely responds on approach, 2=responds to touch, 3=unresponsive to touch (See [17])**

Light Sheet Microscope

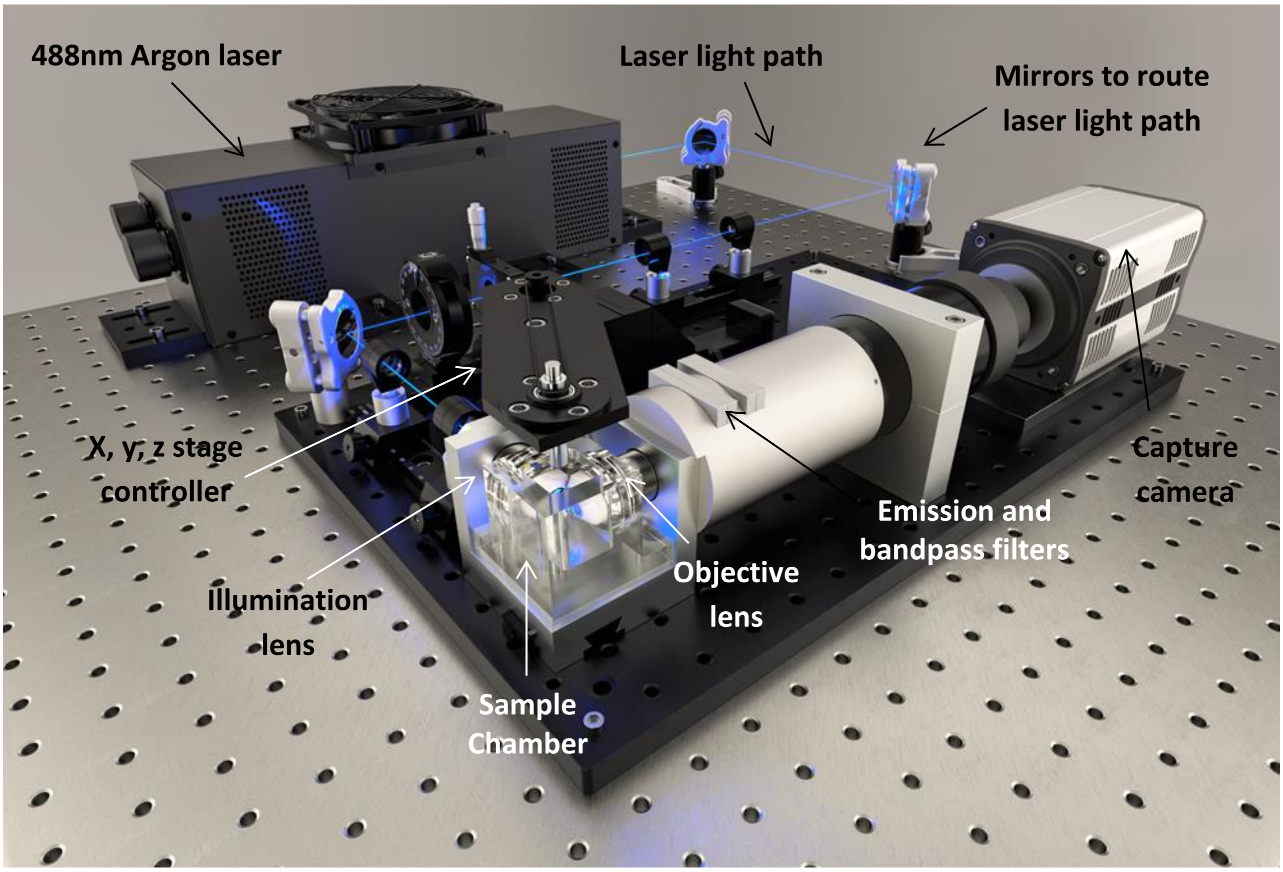

Supplementary Figure 1| Image of the light sheet microscope system used in the current study. Annotations show the main components of the system.

Supplementary Table 2| For reference, the table below shows a list of the 45 anatomical regions used during the registration of anatomical regions across all LSM image sets in the current study. Brain region labels and coding for 3D registration were obtained from the online Z-Brain atlas at http://engertlab.fas.harvard.edu/Z-Brain/downloads.html.

Supra region	Sub region
**Telencephalon**	Telencephalon
	Anterior commissure
	Olfactory bulb
	Pallium
	Subpallium
**Diencephalon**	Diencephalon
	Dorsal thalamus
	Eminentia thalami
	Habenulae
	Intermediate hypothalamus
	Caudal hypothalamus
	Pineal
	Posterior tuberculum
	Preoptic area
	Pretectum
	Rostral hypothalamus
	Ventral thalamus
**Diencephalon**	Mesencephalon
	Tectum stratum periventriculare
	Tectum neuropil
	Tegmentum
	Torus longitudinaris
	Torus semicircularis
**Rhombencephalon**	Rhombencephalon
	Area postrema
	Cerebellum
	Corpus cerebelli
	Eminentia granularis
	Inferior olive
	Interpenduncular nucleus
	Lateral reticular nucleus
	Lobus caudalis cerebelli
	Locus coeruleus
	Mauthner
	Medial vestibular nucleus
	Noradrendergic neurons
	Raphe – inferior
	Raphe – superior
	Tangential vestibular nucleus
	Valvula cerebelli
**Ganglia**	Eyes
	Ofactory epithelium
	Vagal ganglia
	Spinal cord
	Spinal cord neuropil region

***In vivo* electrophysiology**

All electrophysiological recording used 4dpf *elavl3*:GCaMP6s zebrafish. Larvae were immobilised by continuous exposure to 4mM tubocurarine hydrochloride pentahydrate (Tubocurarine; Sigma-Aldrich, Poole, UK) to ensure stability of the zebrafish for experimental recording. The larvae were then fixed and positioned in the recording chamber in 50µl 1% low melting point (LMP; Sigma-Aldrich, Poole, UK) agarose containing extracellular solution (ECS) composed of (in mM): 1 NaCl, 2.9 KCl, 10 HEPES, 1.2 $MgCl_2$, 2.1 $CaCl_2$ and 10 glucose, pH 7.3 NaOH. Zebrafish were positioned dorsal side up, to better expose the brain for electrode placement. Using a Scientifica infra-red microscope 4x magnification (Scientifica, Sussex, UK) under direct visual guidance using ThorCam software (ThorLabs, NJ, USA), a glass microelectrode (<1.2mm tip diameter, 3-5 MΩ) filled with ECS was placed into the optic tectum (**Supplementary Figure 2**), the largest midbrain structure, to record extracellular local field potential from small networks of neurons. Voltage recordings were low-pass filtered at 0.1Hz, high-pass filtered at 200Hz, amplified at a gain of 100x and digitised at 1kHz (MultiClamp 700B amplifier, Digidata 1550 digitiser, Axon Instruments, USA). Zebrafish were equilibrated in 20µl ECS for 600s prior to data acquisition to ensure settling of glass electrode placement and to establish a baseline response. Local field potential (LFP) recording protocols allowed for a 300s baseline period before drug addition to the ECS and experimental recording lasted for a total of 4200s. During all experimentation on larval zebrafish, the heart rate was monitored visually to confirm survival. Only experiments conducted with a visible heartbeat throughout were included in the analysis.

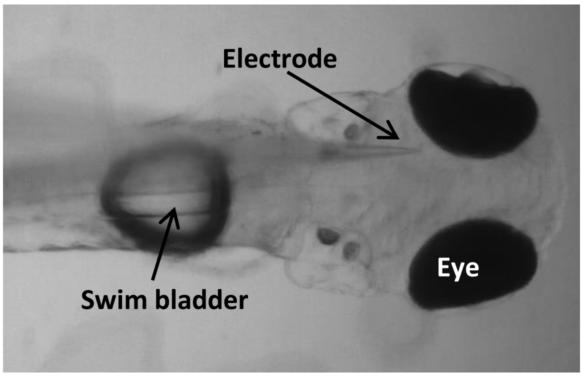

Supplementary Figure 2| Placement of glass electrode in the optic tectum of GCaMP6s:*elavl3* zebrafish larvae. Immobilised 4 dpf *elavl3*:GCaMP6s larval zebrafish fixed and positioned in 1% LMP agarose dorsal side up to facilitate glass electrode access for placement in the optic tectum. The transparent nature of *elavl3*:GCaMP6s zebrafish permits precise electrode placement in specific central nervous system (CNS) structures.

Electrophysiology data were analysed using MATLAB (MathWorks, Cambridge, UK). Temporal profile analysis of experimental recording was performed using the Chronux toolbox (http//:chronux.org/). The frequency of neuronal network events was calculated within the range of 0.1-6.0Hz, this relatively low frequency band was chosen due to the slow nature of neuronal network activity. The power of these events was then calculated within the low frequency band and measured in mV^2. Quantification of the peak frequency and power across all experiments were plotted in 300s epochs. Student's t-tests were performed on paired data using Origin 2016 (OriginLab, MA, USA). Comparisons of the frequency and power of neuronal network activity was made before (0-900s) and after drug addition (3300-4200s) with statistical significance defined as $P<0.05$.

Supplementary Results

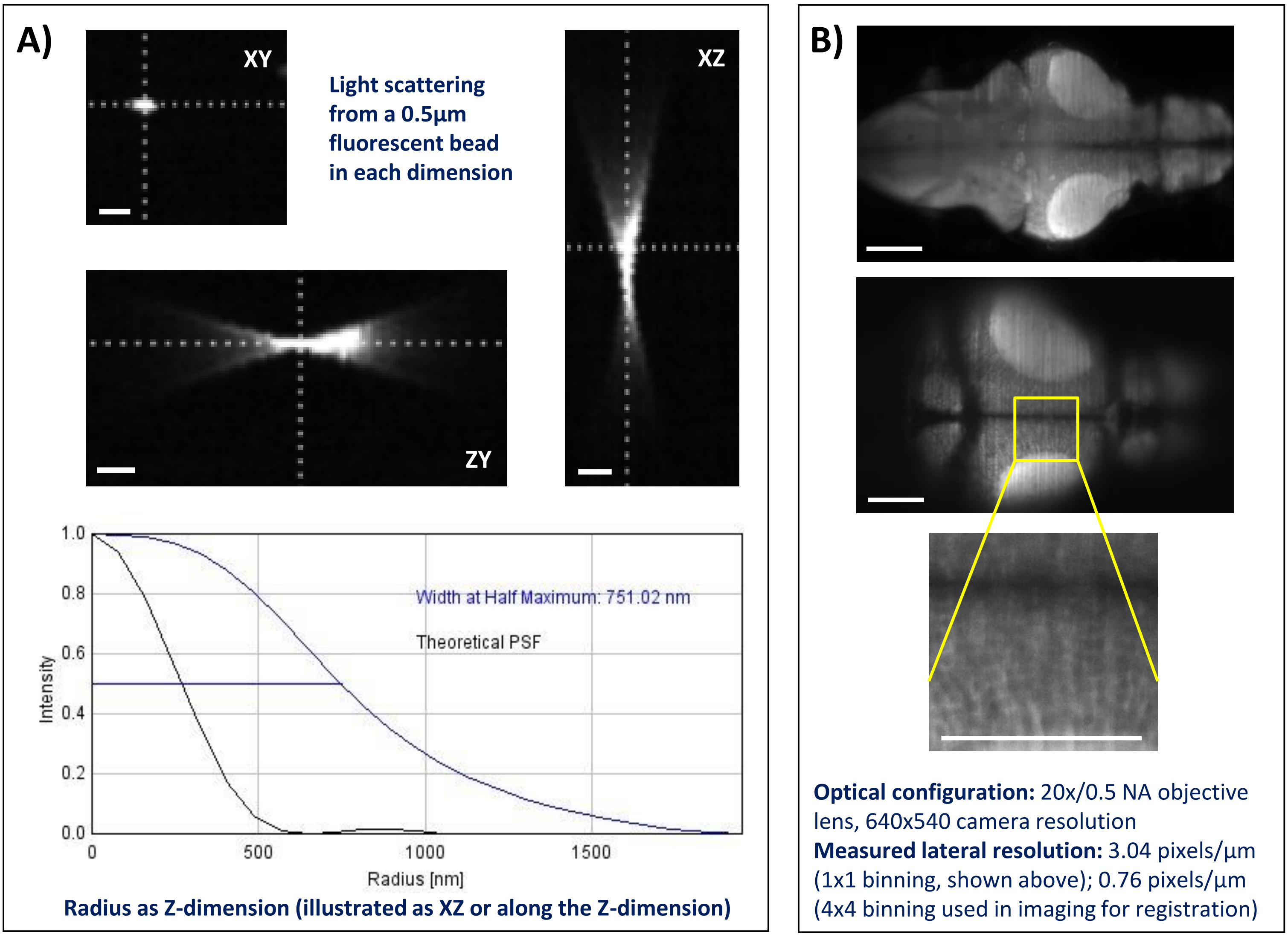

Supplementary Figure 3| Summary of achievable 3D and spatial resolution using the LSM system set up used for whole brain neuropharmacological profiling. Panel **A)** shows the Point Spread Function (PSF) estimation averaged across 50 x 0.5µm fluorescent beads (Emission wavelength: 525nm, Merck Milipore, Haarlerbergweg, The Netherlands) in a 1.5µm step scan using MOSAICsuite (http://imagej.net/MOSAICsuite). The scale bar represents circa. 10µm. Panel **B)** shows a diagrammatic representation of the spatial resolution achievable with the LSM settings used in the current study. Shown are a maximum intensity projection of the whole scan area (top); resolution in a single slice at the top of this stack (middle); and detail from this slice (bottom) in which individual cell bodies are clearly visible. The scale bar represents circa. 100µm. Images were obtained on the 5.5MP sCMOS camera used throughout the study (30FPS, 640x540 pixels, 4x4 binning, 40ms exposure).

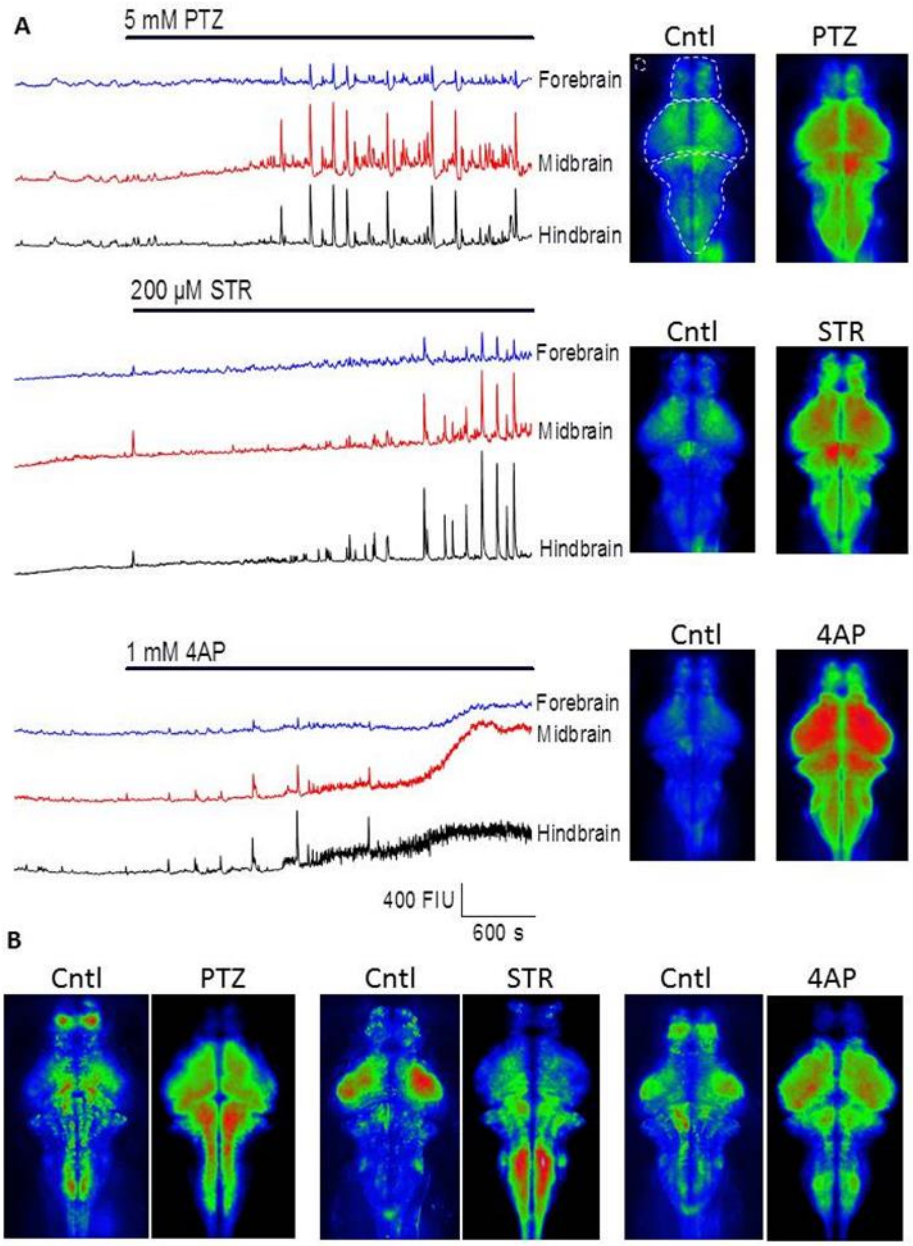

Supplementary Figure 4| Wide-field imaging reveals CNS region-specific convulsant induced hyperactivity **A)** Left, time courses of GCaMP fluorescence intensity from time series collected with wide-field fluorescence imaging in three typical experiments. The bars indicate the time of drug application for PTZ, Strychnine and 4-AP and the portion of the trace to the left hand side of these bars indicates the pre-treatment (baseline) level of activity. The basal level of fluorescence was ~200 fluorescence intensity units in each area and the traces have been offset for clarity. Right, mean intensity projections across 300 consecutive frames (i.e. 5 min) collected either just prior to drug addition (**left**), or after 40-45 minutes drug treatment (**right**). The upper left image illustrates where the forebrain, midbrain and hindbrain regions of interest were located. The corresponding traces are coloured blue, red and black in each of the time course plots. **B)** Plots to indicate the degree of signal variance seen across the imaged x-y plane. These were compiled by creating projections of standard deviation across 300 frames and then (because SD is higher in the drug treated time-points) normalizing each pixel to the average standard deviation across the entire CNS. These normalized images were then pseudo-coloured and displayed between 1/3rd of mean SD (blue) and 3 times mean SD (red). Note how the drugs cause areas of greatest variation to change relative to their in subject controls and also how the different drugs seem to create different patterns.

Supplementary Table 3|SEM values for the data contained in **Figure 4 of the main manuscript**. These data comprise each median (across the whole imaging duration) voxel intensity value, averaged across all fish in that treatment group, expressed a % change versus the corresponding control fish group, within each registered brain region. Control data are shown as the SEM for each region, averaged across the 4 untreated control larval groups. N=8 larvae were imaged per group.

Median data	SEM of % change, control versus treated				
Region of Interest	Mean control	4AP	PTZ	Pilocarpine	Strychnine
**Telencephalon**	1.10	379.17	41.15	28.51	47.54
Anterior commissure	5.48	140.42	24.08	15.72	28.08
Olfactory bulb	1.27	531.11	28.37	8.06	17.81
Pallium	1.01	526.29	60.38	25.56	46.74
Subpallium	0.37	489.78	245.09	88.73	95.22
**Diencephalon**	2.02	218.08	25.12	11.89	59.60
Dorsal thalamus	2.80	208.37	21.93	11.17	73.18
Eminentia thalami	1.99	182.71	17.32	9.49	47.13
Habenulae	1.13	404.01	59.64	24.33	44.61
Intermediate hypothalamus	5.04	180.37	22.41	22.88	34.33
Caudal hypothalamus	n/a	n/a	n/a	n/a	n/a
Pineal	0.27	162.08	24.44	11.37	54.21
Posterior tuberculum	2.74	182.71	34.00	10.84	68.15
Preoptic area	1.54	160.31	31.09	13.11	58.37
Pretectum	3.13	231.55	24.47	10.61	67.35
Rostral hypothalamus	N/A	239.74	30.05	10.86	n/a
Ventral thalamus	2.89	160.88	27.36	11.71	70.50
**Mesencephalon**	3.00	298.13	13.03	12.35	34.39
Tectum stratum periventriculare	2.49	276.96	13.12	12.94	30.16
Tectum neuropil	3.39	452.76	13.33	13.21	40.34
Tegmentum	5.54	160.77	12.90	13.82	37.03
Torus longitudinaris	0.55	370.45	29.04	12.80	43.99
Torus semicircularis	3.70	302.91	14.31	14.85	39.28
**Rhombencephalon**	4.09	148.76	10.71	12.37	36.70
Area postrema	3.87	149.93	29.18	22.15	39.95
Cerebellum	2.91	210.06	13.75	10.54	45.92
Corpus cerebelli	3.14	206.41	14.42	11.50	46.50
Eminentia granularis	3.11	200.45	11.31	18.20	36.66
Inferior olive	7.69	96.44	13.16	15.51	18.11
Interpenduncular nucleus	6.56	129.72	15.02	18.77	20.66
Lateral reticular nucleus	3.23	244.63	11.74	15.33	27.35
Lobus caudalis cerebelli	1.50	245.02	20.95	11.87	23.73
Locus coeruleus	6.52	114.30	14.39	16.85	37.16
Mauthner	4.97	115.89	12.73	14.87	47.81
Medial vestibular nucleus	6.01	138.89	14.40	13.43	38.72
Noradrendergic neurons	4.38	237.29	30.92	13.90	30.80
Raphe – inferior	5.88	99.30	11.12	14.49	34.72
Raphe – superior	5.76	130.12	12.20	13.05	41.80
Tangential vestibular nucleus	3.59	152.26	14.55	13.58	43.42
Valvula cerebelli	2.63	227.92	15.55	11.69	51.42
Eyes	0.15	53.87	5.93	6.64	24.67
Ofactory epithelium	0.15	115.38	15.25	7.84	17.71
Vagal ganglia	1.50	122.53	11.97	21.72	18.31
Spinal cord	3.13	85.63	13.73	12.97	16.37
Spinal cord neuropil region	5.05	94.34	13.00	13.24	16.93

Supplementary Table 4| Statistical comparisons of the time-averaged median fluorescence intensity values obtained per region, averaged across all fish in each treatment, versus the control group value. Analysis was undertaken using the Kruskal Wallis test followed by Dunn's post hoc comparison of control and treated groups within each region. Only data where a significant difference versus the corresponding control group are shown. Data shown are the overall statistical significance (K-W test) and significant increases (↑) or decrease (↓) at the p<0.05 (*), P<0.01 (**) or P<0.001 (***) level for that treatment group versus the corresponding control group. N=8 larvae were imaged per group.

Compound	Brain region	KW-test	Versus control group		
			low	Medium	High
4AP	Torus semicircularis	P<0.05		↑*	↑*
	Mauthner cells	P<0.05		↑*	↑*
	Medial vestibular nucleus	P<0.05			↑**
	Tangential vestibular nucleus	P<0.05		↑**	↑**
PTZ	Eminentia thalami	P<0.05			↓**
	Preoptic area	P<0.05			↓**
	Tectum stratum periventriculare	P<0.05	-	-	-
	Lobus caudalis cerebelli	P<0.05	-	-	-
	Spinal cord	P<0.05			↓**
	Olfactory Bulb	P<0.05			↑**
Pilocarpine	Area postrema	P<0.05		↓*	
	Noradrendergic neurons of the Interfascicular and Vagal areas	P<0.01		↓**	↓*
	Raphe – Inferior	P<0.05		↓*	
	Spinal cord	P<0.05		↓**	
Strychnine	No significant differences	-	-	-	-

A) Area under the Curve (AUC)	Control	% Change, control versus treated			
Region of Interest	mean	4AP/10	PTZ	Pilocarpine	Strychnine
**Telencephalon**	230.66	440.25	-0.37	21.34	-25.14
Anterior commissure	274.24	293.68	30.17	-49.14	208.01
Olfactory bulb	404.50	357.76	15.27	140.17	-81.22
Pallium	142.78	460.52	-13.82	55.10	39.95
Subpallium	237.25	149.43	185.92	328.74	2.41
**Diencephalon**	200.34	342.62	42.86	110.37	249.73
Dorsal thalamus	307.08	349.33	7.42	42.72	271.50
Eminentia thalami	291.23	187.46	-55.81	-39.84	39.73
Habenulae	208.10	214.20	51.09	-37.34	-34.60
Intermediate hypothalamus	312.73	385.19	54.16	31.77	124.19
Caudal hypothalamus	301.78	563.34	n/a	n/a	n/a
Pineal	30.90	541.51	120.33	11.88	93.54
Posterior tuberculum	206.15	264.49	31.17	44.69	229.89
Preoptic area	149.07	215.44	-31.48	20.23	80.68
Pretectum	292.34	336.22	96.46	40.55	253.02
Rostral hypothalamus	129.31	287.08	16.18	-0.81	3.04
Ventral thalamus	295.44	251.44	-3.19	81.39	260.98
**Mesencephalon**	275.37	539.29	81.17	41.29	207.30
Tectum stratum periventriculare	312.37	432.37	-2.54	82.36	112.48
Tectum neuropil	447.71	591.50	3.81	102.36	50.77
Tegmentum	423.43	326.33	77.13	23.95	348.05
Torus longitudinaris	90.33	724.37	3.15	-22.12	20.96
Torus semicircularis	322.71	472.48	86.98	42.76	253.24
**Rhombencephalon**	281.85	406.53	79.63	-12.36	290.25
Area postrema	204.73	253.31	14.95	-29.33	124.25
Cerebellum	226.02	453.73	103.71	0.15	355.44
Corpus cerebelli	246.61	444.28	108.55	-1.29	371.89
Eminentia granularis	309.75	494.55	29.48	-50.79	234.05
Inferior olive	439.97	267.26	9.83	-10.59	65.75
Interpenduncular nucleus	496.39	248.72	67.46	121.39	54.32
Lateral reticular nucleus	208.87	612.11	31.56	-25.65	100.70
Lobus caudalis cerebelli	213.72	424.72	131.88	-62.23	97.69
Locus coeruleus	460.27	299.09	98.86	8.79	356.77
Mauthner	385.63	346.03	73.44	-2.96	398.97
Medial vestibular nucleus	373.48	436.78	114.33	-26.13	349.05
Noradrendergic neurons	166.83	413.76	49.23	-58.25	200.12
Raphe – inferior	395.02	282.35	47.60	-7.86	299.88
Raphe – superior	500.84	245.33	54.05	18.83	277.98
Tangential vestibular nucleus	316.82	247.36	59.03	-3.91	150.89
Valvula cerebelli	220.31	480.51	118.73	-25.75	450.89
Eyes	55.60	412.94	-9.16	100.07	89.91
Ofactory epithelium	43.46	238.14	120.76	-22.71	-44.05
Vagal ganglia	231.63	202.28	5.20	-69.08	58.72
Spinal cord	232.10	199.14	-2.90	-62.57	44.45
Spinal cord neuropil region	293.74	229.55	-8.70	-34.41	22.95

B) Peak intensity	Control	% Change, control versus treated			
Region of Interest	mean	4AP/10	PTZ	Pilocarpine	Strychnine
**Telencephalon**	16.76	182.05	13.10	21.21	8.54
Anterior commissure	62.27	91.11	35.98	-50.90	150.05
Olfactory bulb	22.67	178.54	21.13	12.34	-69.94
Pallium	12.66	204.40	11.07	27.07	48.76
Subpallium	66.19	49.44	142.97	206.25	-4.63
**Diencephalon**	26.33	156.09	61.54	3.51	140.00
Dorsal thalamus	30.48	150.84	61.77	8.48	190.77
Eminentia thalami	22.28	119.68	-36.75	-19.83	40.88
Habenulae	12.26	184.12	70.44	-0.80	-11.23
Intermediate hypothalamus	63.36	128.84	65.07	28.02	117.60
Caudal hypothalamus	61.64	170.46	n/a	n/a	n/a
Pineal	3.93	169.85	39.00	3.61	110.51
Posterior tuberculum	28.60	107.65	29.07	-11.58	101.77
Preoptic area	13.38	110.75	-25.76	-12.24	84.97
Pretectum	33.32	152.24	116.64	11.15	170.56
Rostral hypothalamus	18.04	147.43	17.12	-5.12	-23.05
Ventral thalamus	29.32	111.95	60.23	-2.99	179.15
**Mesencephalon**	34.52	249.63	92.32	-2.99	162.36
Tectum stratum periventriculare	27.75	267.22	54.57	-6.41	106.38
Tectum neuropil	38.42	320.76	53.61	6.03	105.38
Tegmentum	80.44	111.68	103.29	-7.50	211.98
Torus longitudinaris	7.10	316.76	45.19	-11.97	50.55
Torus semicircularis	36.75	278.01	72.21	-7.98	231.90
**Rhombencephalon**	58.90	123.55	87.83	-26.83	206.25
Area postrema	37.92	101.85	15.19	-53.26	118.56
Cerebellum	38.33	184.52	109.31	-20.65	219.58
Corpus cerebelli	41.81	180.80	119.11	-23.74	223.56
Eminentia granularis	44.89	166.14	40.69	-18.43	163.85
Inferior olive	100.77	73.78	21.18	-18.32	55.44
Interpenduncular nucleus	72.15	115.70	73.59	14.22	109.52
Lateral reticular nucleus	44.56	211.22	28.66	-39.86	79.87
Lobus caudalis cerebelli	24.99	191.64	106.75	-43.92	121.25
Locus coeruleus	104.32	80.92	90.77	-16.01	230.20
Mauthner	83.62	106.23	83.47	-20.65	267.65
Medial vestibular nucleus	84.26	132.90	96.74	-36.51	236.18
Noradrendergic neurons	36.14	162.17	47.47	-64.91	154.73
Raphe – inferior	86.94	87.51	52.07	-21.78	212.05
Raphe – superior	68.87	107.25	110.30	-0.82	230.27
Tangential vestibular nucleus	45.50	131.69	68.22	-16.32	192.91
Valvula cerebelli	30.76	222.42	132.66	-32.17	251.66
Eyes	5.77	194.01	22.33	12.81	94.10
Ofactory epithelium	3.44	112.36	27.88	-18.54	-23.82
Vagal ganglia	15.40	132.53	-9.08	-50.05	103.46
Spinal cord	45.04	64.23	2.01	-49.90	26.10
Spinal cord neuropil region	67.62	70.38	3.10	-41.58	15.31

**C) Number of Peaks**	**Control**	**% Change, control versus treated**			
**Region of Interest**	**mean**	**4AP**	**PTZ**	**Pilocarpine**	**Strychnine**
**Telencephalon**	5.23	-31.71	4.76	14.63	16.67
Anterior commissure	7.97	-38.89	-29.73	33.85	-35.90
Olfactory bulb	6.78	-3.17	-17.19	-15.69	31.71
Pallium	5.05	-19.44	31.25	10.26	2.56
Subpallium	4.13	-31.25	20.00	-10.00	37.93
**Diencephalon**	6.97	-49.12	-34.33	-16.00	-16.13
Dorsal thalamus	5.58	-53.57	-14.89	-22.50	-13.33
Eminentia thalami	5.50	-17.14	26.19	40.00	4.35
Habenulae	5.37	-8.11	-10.26	46.88	0.00
Intermediate hypothalamus	7.31	-56.06	-32.26	-24.00	-41.46
Caudal hypothalamus	3.63	-95.24	n/a	n/a	n/a
Pineal	8.76	-43.02	-18.67	1.23	-14.75
Posterior tuberculum	7.17	-51.72	-42.86	-9.30	-20.31
Preoptic area	5.46	-37.04	-1.92	0.00	6.00
Pretectum	5.58	-40.82	-20.83	-25.64	-18.64
Rostral hypothalamus	4.23	-31.25	-66.67	140.00	-88.89
Ventral thalamus	5.98	-46.30	-10.87	-31.43	-22.95
**Mesencephalon**	6.27	-66.04	-40.30	-1.82	-31.58
Tectum stratum periventriculare	6.64	-56.52	-18.00	-17.02	-12.50
Tectum neuropil	6.41	-63.89	-31.25	-32.00	14.29
Tegmentum	7.24	-50.00	-36.11	-3.95	-36.71
Torus longitudinaris	6.67	-28.99	18.52	15.63	2.78
Torus semicircularis	7.89	-48.08	-51.81	3.57	-22.39
**Rhombencephalon**	8.76	-65.28	-46.34	7.50	-39.02
Area postrema	6.51	-33.87	-17.74	-1.64	-30.51
Cerebellum	8.82	-56.14	-42.86	1.45	-40.96
Corpus cerebelli	8.71	-55.36	-40.54	0.00	-40.24
Eminentia granularis	7.44	-60.61	-42.17	10.29	-30.30
Inferior olive	7.14	-44.44	-42.86	6.76	-40.26
Interpenduncular nucleus	7.47	-36.07	-29.33	-29.73	-18.03
Lateral reticular nucleus	8.04	-34.78	-39.56	21.33	-40.00
Lobus caudalis cerebelli	5.88	-37.93	-34.29	3.08	-36.76
Locus coeruleus	9.53	-49.32	-42.68	-4.55	-45.45
Mauthner	9.63	-60.00	-41.67	-3.53	-45.98
Medial vestibular nucleus	8.92	-63.75	-44.32	6.25	-40.70
Noradrendergic neurons	8.84	-37.50	-26.92	19.72	-42.17
Raphe – inferior	9.18	-47.06	-47.13	-6.98	-47.06
Raphe – superior	8.22	-47.27	-19.64	-7.81	-20.31
Tangential vestibular nucleus	9.50	-51.67	-41.18	-15.58	-36.71
Valvula cerebelli	5.97	-53.33	-44.16	21.43	-35.06
Eyes	6.52	-42.00	-12.70	-12.96	16.67
Ofactory epithelium	8.05	-31.65	-31.11	4.48	-9.43
Vagal ganglia	6.48	-70.15	1.56	69.44	3.51
Spinal cord	7.37	-35.48	-37.14	50.00	-37.14
Spinal cord neuropil region	6.21	-36.76	-36.84	26.98	-38.96

D) Peak width	Control	% Change, control versus treated			
Region of Interest	mean	4AP	PTZ	Pilocarpine	Strychnine
**Telencephalon**	14.33	56.23	-22.08	2.78	-35.65
Anterior commissure	5.19	120.30	11.79	9.43	3.61
Olfactory bulb	9.77	62.05	-5.00	69.23	-14.81
Pallium	14.93	23.42	-33.87	13.14	-11.55
Subpallium	4.01	116.11	-1.97	20.94	24.75
**Diencephalon**	9.23	95.19	-13.27	80.17	-9.07
Dorsal thalamus	12.95	85.56	-33.01	20.46	-13.40
Eminentia thalami	16.24	13.26	-14.03	-31.33	-8.83
Habenulae	15.65	16.31	-10.34	-39.39	-10.72
Intermediate hypothalamus	5.92	165.84	-0.29	-3.83	-9.75
Caudal hypothalamus	5.50	260.82	n/a	n/a	n/a
Pineal	8.21	124.98	38.07	8.48	-0.81
Posterior tuberculum	9.14	115.87	2.19	45.43	-8.18
Preoptic area	14.62	39.76	-12.83	32.29	-31.72
Pretectum	10.89	58.57	-8.37	16.82	-9.62
Rostral hypothalamus	8.52	70.99	5.79	-11.86	45.02
Ventral thalamus	12.77	88.89	-37.51	71.45	-15.63
**Mesencephalon**	9.12	98.14	-2.45	27.74	-8.50
Tectum stratum periventriculare	12.45	45.29	-33.82	79.92	-17.63
Tectum neuropil	11.52	75.90	-19.98	62.57	-35.38
Tegmentum	6.29	127.54	-4.61	22.65	11.07
Torus longitudinaris	11.96	55.10	-18.62	-10.47	-12.32
Torus semicircularis	10.32	41.05	15.99	32.07	-20.56
**Rhombencephalon**	5.50	139.01	11.24	18.09	4.64
Area postrema	6.77	69.58	8.80	24.28	13.41
Cerebellum	7.29	78.38	0.86	13.03	13.09
Corpus cerebelli	7.47	75.65	-1.71	8.57	11.20
Eminentia granularis	7.58	151.24	3.07	-40.74	-3.08
Inferior olive	5.00	117.45	11.72	6.51	10.36
Interpenduncular nucleus	7.83	99.51	1.39	59.19	-30.87
Lateral reticular nucleus	5.52	90.59	8.84	26.79	-7.55
Lobus caudalis cerebelli	8.42	94.64	15.56	-27.94	-20.56
Locus coeruleus	5.04	142.43	17.98	27.02	17.99
Mauthner	5.21	158.22	8.27	20.63	12.31
Medial vestibular nucleus	5.12	166.31	23.48	17.10	4.88
Noradrendergic neurons	5.96	60.88	10.37	22.68	12.83
Raphe – inferior	5.16	114.31	16.52	21.38	9.96
Raphe – superior	8.48	103.75	-25.35	9.74	-9.62
Tangential vestibular nucleus	7.10	76.04	11.28	12.05	-22.55
Valvula cerebelli	8.73	72.03	-5.27	-10.82	23.31
Eyes	10.37	81.46	-14.88	70.70	-20.96
Ofactory epithelium	9.25	47.44	65.61	-8.36	2.97
Vagal ganglia	12.11	70.44	9.58	-42.66	-13.80
Spinal cord	5.66	106.01	13.80	-21.86	25.06
Spinal cord neuropil region	4.87	115.89	5.20	10.11	7.00

E) Peak separation	Control	% Change, control versus treated			
Region of Interest	mean	4AP	PTZ	Pilocarpine	Strychnine
**Telencephalon**	29.51	-7.29	-9.36	1.91	-11.62
Anterior commissure	20.53	84.61	0.30	40.20	2.60
Olfactory bulb	25.25	-19.31	116.89	3.34	-36.33
Pallium	30.67	6.51	34.07	-9.10	-8.65
Subpallium	38.58	5.55	2.81	36.13	-2.58
**Diencephalon**	24.83	26.35	-19.56	58.20	84.20
Dorsal thalamus	27.55	46.75	75.00	6.73	67.22
Eminentia thalami	31.60	1.66	20.99	7.95	10.65
Habenulae	29.55	44.20	1.98	7.62	-23.16
Intermediate hypothalamus	19.71	56.79	11.19	63.41	14.77
Caudal hypothalamus	17.64	n/a	n/a	n/a	n/a
Pineal	21.64	-6.48	3.08	25.92	-19.50
Posterior tuberculum	24.26	52.09	18.25	67.68	60.64
Preoptic area	27.20	16.94	34.75	39.90	-0.06
Pretectum	25.54	55.62	22.35	74.55	16.70
Rostral hypothalamus	20.91	140.92	72.73	163.60	95.78
Ventral thalamus	28.97	61.71	43.27	7.87	52.74
**Mesencephalon**	22.48	71.03	19.40	167.91	24.33
Tectum stratum periventriculare	26.11	48.40	20.28	24.65	67.46
Tectum neuropil	27.53	10.33	29.46	152.02	-10.48
Tegmentum	21.00	88.23	11.80	51.51	-15.12
Torus longitudinaris	31.94	-32.60	-20.36	-5.90	33.52
Torus semicircularis	22.85	44.66	42.77	125.61	-13.68
**Rhombencephalon**	19.19	93.56	3.23	64.79	56.15
Area postrema	22.02	80.37	5.94	26.31	-13.04
Cerebellum	21.48	102.93	12.38	63.64	-1.90
Corpus cerebelli	21.45	99.19	1.49	75.37	-6.24
Eminentia granularis	21.38	77.05	7.31	56.08	50.50
Inferior olive	20.03	89.09	2.15	58.32	13.06
Interpenduncular nucleus	21.94	29.77	87.57	34.53	-12.09
Lateral reticular nucleus	19.88	91.94	4.88	48.34	-10.50
Lobus caudalis cerebelli	20.19	86.53	24.76	38.96	-23.54
Locus coeruleus	18.86	104.55	10.73	49.11	45.70
Mauthner	18.76	103.93	9.09	53.68	7.72
Medial vestibular nucleus	18.37	96.53	9.76	77.75	68.16
Noradrendergic neurons	20.50	97.38	3.76	53.00	-6.66
Raphe – inferior	18.78	102.79	21.79	60.89	33.79
Raphe – superior	22.07	82.94	41.79	45.34	-9.97
Tangential vestibular nucleus	21.18	69.99	30.50	50.36	3.16
Valvula cerebelli	21.25	86.81	-4.54	77.62	79.85
Eyes	26.29	56.22	8.45	9.46	26.87
Ofactory epithelium	20.86	30.76	1.24	32.25	-0.26
Vagal ganglia	25.41	10.65	22.24	16.66	18.68
Spinal cord	21.65	55.40	-9.69	67.29	23.08
Spinal cord neuropil region	20.07	64.39	-3.38	70.05	16.80

Supplementary Figure 5| Summary of the data in **Figure 5 of the main manuscript**. Data shown are the peak profile analysis results for each region, across the 4 treatments. The treatment group shown is that at which the highest activity was observed (top concentration for all except 2.5mM for PTZ due to a slight dip at the highest treatment level). Each data point is the mean across all fish in that treatment group expressed as the % change versus the mean of the corresponding control group $(F_1-F_0)/F_0*100$, where F_1= treated group value, and F_0= control group value. Note that for the AUC and the peak height profiles, 4AP data were divided by 10 to allow plotting on the same axis. For brevity control data shown as the mean of the 4 untreated control groups. Colour coding represents the degree of activation (shades of red), or suppression (shades of blue) versus that region in the control larvae, relative to other brain regions within that treatment group. n/a – values not obtained. N=8 larvae were imaged per group.

Supplementary Table 5| Results of the statistical comparison of the temporal profile analysis. For each parameter, data were averaged across all fish in each treatment, and the resultant values compared, on a region by region basis, with the control group value. Statistical comparisons were undertaken using the Kruskal Wallis test followed by Dunn's post hoc comparison of control and treated groups. Only data where a significant difference versus the corresponding control group are shown. Data shown are the overall statistical significance (K-W test) and significant increases (↑) or decrease (↓) at the p<0.05 (*), P<0.01 (**) or P<0.001 (***) level for that treatment group versus the corresponding control group. N=8 larvae were imaged per group.

Compound	Temporal profile parameter	Brain region	KW-test	Versus control group		
				low	Med	High
4AP	AUC	Tectum stratum periventriculare	P<0.05		↑**	
		Tectum neuropil	P<0.05		↑*	↑**
		Emenetia granularis	P<0.05		↑*	↑*
		Mauthner	P<0.05		↑*	↑*
		Medial vestibular nucleus	P<0.05			↑**
		Tangential vestibular nucleus	P<0.05		↑*	↑**
		Valvular cerebelli	P<0.05		↑**	↑*
		Vagal ganglia	P<0.05		↑*	↑**
		Mesencephalon	P<0.05		↑*	↑*
4AP	Peak height	Tectum stratum periventriculare	P<0.05		↑**	
		Tectum neuropil	P<0.05		↑**	↑*
		Mauthner	P<0.05		↑*	
		Medial vestibular nucleus	P<0.05		↑*	↑*
		Tangential vestibular nucleus	P<0.05		↑**	↑**
		Vagal ganglia	P<0.05		↑**	↑**
		Mesencephalon	P<0.05		↑*	
4AP	Peak number	Tectum stratum periventriculare	P<0.05		↓*	↓**
		Tectum neuropil	P<0.05			↓**
		Rhombencephalon	P<0.05			↓**
		Emenetia granularis	P<0.05			↓**
		Mauthner	P<0.05		↓**	↓**
		Medial vestibular nucleus	P<0.05			↓**
		Valvulae cerebelli	P<0.05		↓**	
		Vagal ganglia	P<0.05			↓**
		Mesencephalon	P<0.05			↓**
4AP	Peak separation	No differences detected				
4AP	Peak width	Tectum neuropil	P<0.05	-	-	-
		Dorsal thalamus	P<0.05			↑**
		Eminentia granularis	P<0.05			↑**
		Intermediate hypothalamus	P<0.05			↑**
		Ventral thalamus	P<0.05			↑**
		Mesencephalon	P<0.05			↑**

Compound	Temporal profile parameter	Brain region	KW-test	Versus control group		
				low	Med	High
PTZ	AUC	Cerebellum	P<0.05		↑**	
		Corpus cerebelli	P<0.05		↑**	
		Lobus caudalis cerebelli	P<0.05		↑*	
		Locus coeruleus	P<0.05		↑*	
		Medial Vestibular nucleus	P<0.05		↑**	
		Valvula cerebelli	P<0.05		↑**	
		Preoptic area	P<0.05			↓**
PTZ	Peak height	Tectum stratum periventriculare	P<0.05		↑*	
		Rhombencephalon	P<0.05		↑**	
		Emenentia thalami	P<0.05			↓**
		Cerebellum	P<0.05		↑**	
		Corpus cerebelli	P<0.05		↑**	
		Lobus caudalis cerebelli	P<0.05		↑**	
		Raphe superior	P<0.01		↑**	
		Valvular cerebelli	P<0.01		↑**	
		Olfactory bulb	P<0.05	↑**		
		Preoptic area	P<0.05			↓**
		Pretectum	P<0.05		↑**	
		Mesencephalon	P<0.05		↑**	
PTZ	Peak number	Tegmentum	P<0.05			↓**
		Torus semicircularis	P<0.001		↓***	↓***
		Rhombencephalon	P<0.01		↓**	↓**
		Cerebellum	P<0.05		↓*	↓**
		Corpus cerebelli	P<0.05			↓**
		Eminentia granularis	P<0.05		↓*	↓**
		Interpenduncular nucleus	P<0.01			↓**
		Lateral reticular nucleus	P<0.01		↓*	↓**
		Lobus caudalis cerebelli	P<0.05			↓*
		Locus coeruleus	P<0.01		↓**	↓**
		Mauthner	P<0.01		↓**	↓**
		Medial vestibular nucleus	P<0.05		↓**	↓**
		Noradrenergic neurons	P<0.05			↓**
		Raphe – Inferior	P<0.05		↓**	↓**
		Tangential vestibular nucleus	P<0.05		↓*	↓*
		Valvula cerebelli	P<0.01		↓**	↓**
		Subpallium	P<0.05	-	-	-
		Posterior tuberculum	P<0.05		↓*	↓*
		Mesencephalon	P<0.05		↓*	
PTZ	Peak separation	Diencephalon	P<0.01		↑*	↑**
		Tectum stratum periventriculare	P<0.05	-	-	-
		Tectum neuropil	P<0.05		↑**	
		Tegmentum	P<0.01			↑***
		Torus semicircularis	P<0.001		↑***	↑***
		Rhombencephalon	P<0.001		↑**	↑***
		Cerebellum	P<0.001		↑**	↑***
		Corpus cerebelli	P<0.01		↑**	↑***
		Eminentia granularis	P<0.01		↑**	↑**
		Inferior olive	P<0.01		↑*	↑***
		Interpenduncular nucleus	P<0.01			↑**
		Lateral reticular nucleus	P<0.01		↑*	↑***
		Lobus caudalis cerebelli	P<0.01			↑***
		Locus coeruleus	P<0.01			↑***
		Mauthner	P<0.01		↑**	↑**
		Medial Vestibular Nucleus	P<0.001		↑**	↑***
		Noradrendergic neurons	P<0.01		↑**	↑***
		Raphe – Inferior	P<0.01		↑**	↑***
		Raphe – Superior	P<0.05			↑*
		Valvula Cerebelli	P<0.001		↑***	↑***
		Spinal cord	P<0.05			↑**
		Neuropil Region	P<0.01		↑**	↑***
		Anterior Commissure	P<0.01			↑**
		Intermediate hypothalamus	P<0.01		↑*	↑***
		Posterior tuberculum	P<0.01		↑*	↑**
		Pretectum	P<0.001		↑**	↑***
		Rostral hypothalamus	P<0.05		↑*	
		Mesencephalon	P<0.01		↑**	↑***
PTZ	Peak width	Tectum stratum periventriculare	P<0.05			↓**
		Torus semicircularis	P<0.05	-	-	-

Compound	Temporal profile parameter	Brain region	KW-test	Versus control group		
				low	Med	High
Pilocarpine	AUC	Area Postrema	P<0.05	-	-	-
		Noradrenergic neurons	P<0.05			↓*
Pilocarpine	Peak height	Tegmentum	P<0.05	↓**		
		Rhombencephalon	P<0.05	↓**		
		Area postrema	P<0.05	-	-	-
		Inferior olive	P<0.05	↓**		
		Interpenduncular nucleus	P<0.05	-	-	-
		Locus coreuleus	P<0.05	↓**		
		Mauthner	P<0.05	↓**		
		Medial vestibular nucleus	P<0.05	↓**		
		Raphe inferior	P<0.05	↓**		
		Raphe superior	P<0.05	↓**		
		Spinal cord neuropil region	P<0.05	↓**		
		Anterior commissure	P<0.05	↓**		
Pilocarpine	Peak number	Eminentia granularis	P<0.05	-	-	-
		Locus coreuleus	P<0.05		↓**	
		Mauthner	P<0.01		↓**	
		Medial vestibular nucleus	P<0.05	-	-	-
		Raphe – Inferior	P<0.05		↓*	
		Tangential vestibular nucleus	P<0.05		↓**	
Pilocarpine	Peak separation	Locus coeruleus	P<0.05		↑**	
		Mauthner	P<0.01		↑**	
		Medial vestibular nucleus	P<0.01		↑**	
		Raphe – Inferior	P<0.05		↑**	
		Intermediate hypothalamus	P<0.05		↑**	
Pilocarpine	Peak width	Tegmentum	P<0.05		↑**	
		Interpenduncular nucleus	P<0.05	-	-	-
		Lateral reticular nucleus	P<0.05		↑**	
		Locus coeruleus	P<0.05	↑**	↑*	
		Mauthner	P<0.05	↑**		
		Raphe – Inferior	P<0.05	↑*		
		Intermediate hypothalamus	P<0.05	-	-	-
		Vagal ganglia	P<0.05	-	-	-

Compound	Temporal profile parameter	Brain region	KW-test	Versus control group		
				low	Med	High
Strychnine	AUC	No differences detected				
Strychnine	Peak height	No differences detected				
Strychnine	Peak number	Cerebellum	P<0.05		↓**	
		Lateral reticular nucleus	P<0.05			↓*
		Mauthner	P<0.05	↓*	↓**	↓**
		Medial vestibular nucleus	P<0.05		↓**	
		Raphe – inferior	P<0.05	↓*		↓*
		Spinal cord neuropil region	P<0.05			↓*
Strychnine	Peak separation	Tegmentum	P<0.05		↑**	↑**
		Rhombencephalon	P<0.05		↑**	↑**
		Cerebellum	P<0.05		↑**	↑**
		Corpus cerebelli	P<0.05		↑**	↑**
		Inferior olive	P<0.05		↑**	↑**
		Lateral reticular nucleus	P<0.05		↑**	↑**
		Lobus caudalis cerebelli	P<0.01		↑***	
		Locus coeruleus	P<0.01		↑**	↑**
		Mauthner	P<0.05		↑**	↑**
		Medial vestibular nucleus	P<0.05		↑**	↑**
		Noradrenergic neurons	P<0.05		↑**	↑*
		Raphe – inferior	P<0.01		↑**	↑**
		Spinal cord	P<0.01	↑**	↑**	↑**
		Spinal cord neuropil region	P<0.01	↑*	↑**	↑**
		Anterior commissure	P<0.01		↑**	↑**
Strychnine	Peak width	Tectum neuropil	P<0.01		↓***	
		Torus semicircularis	P<0.01		↓***	
		Mesencephalon	P<0.05		↓**	

In vivo electrophysiology

Basal neuronal network events recorded from the *elavl3*:GCaMP6s larval zebrafish optic tectum *in vivo* were first characterised by analysing two quantifiable features of spontaneous waveform activity: the frequency (Hz) and power (mV^2) (**Figure 6**). The stability of these waveform properties (**Figure 6C**) across long experimental durations allowed for pharmacological modulation of tectal neuronal network activity in the presence of different neuroactive drugs (**Figures 6-10**).

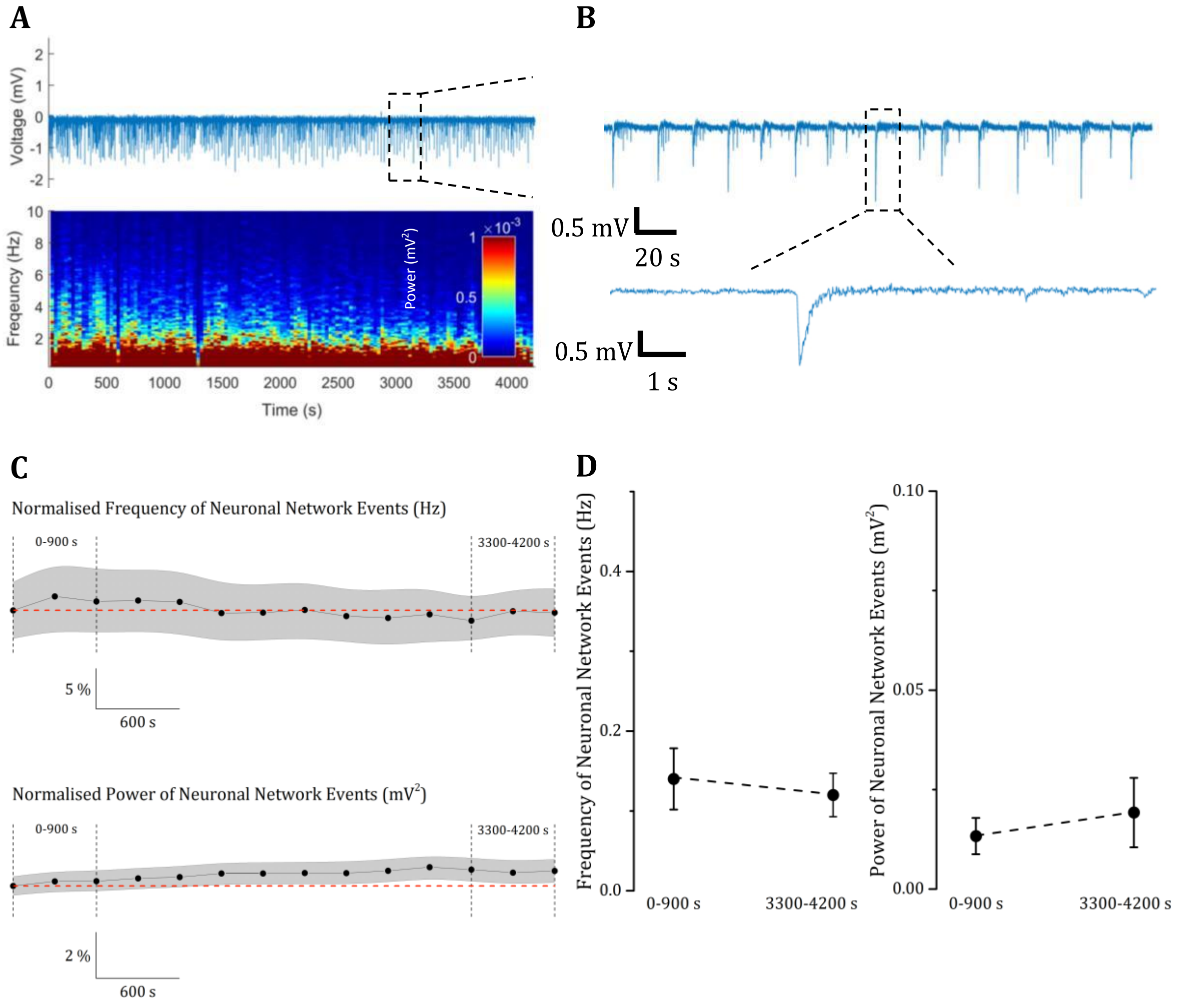

Supplementary Figure 6| Frequency and power of neuronal network events remain stable in *elavl3*:GCaMP6s zebrafish larvae. **A)** Experimental trace and supporting spectrogram of basal waveform activity recorded from the optic tectum of control zebrafish larvae. **B)** Typical spontaneous neuronal network event waveforms, displaying relatively slow and large depolarisations. **C)** The baseline normalised frequency and power of neuronal network events does not change over the time course of the experiment (n=6 zebrafish). The power of neuronal events for all experiments is measured in the 0.2-6.0Hz frequency band. **D)** The frequency (Hz) and power (mV^2) of neuronal network events at the end of the experiment (3300-4200s) is not significantly different to those at the start of the experiment (0-900s) ($P>0.05$, n=6 zebrafish, paired Student's t-test).

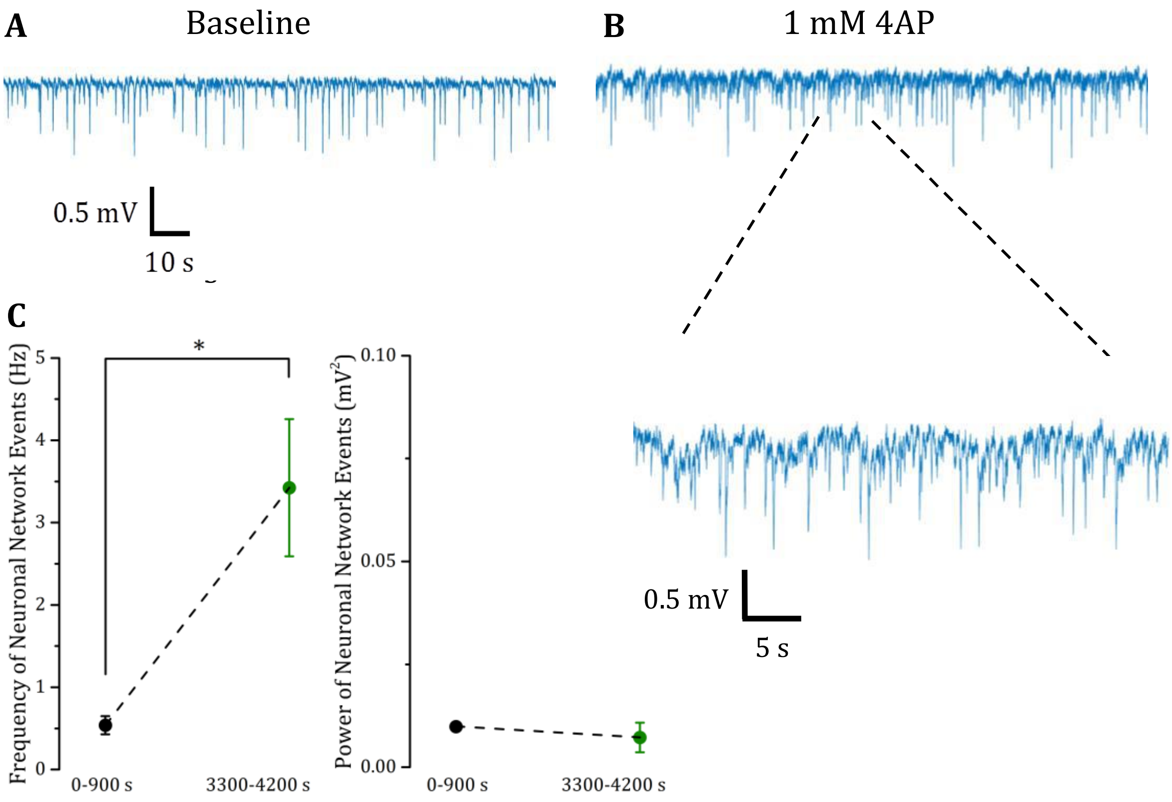

Supplementary Figure 7| 1mM 4AP application shows an increase in frequency of neuronal network events. (A) Representative trace of baseline and 1mM 4AP optic tectum waveform activity. (B) Enhanced electrographic activity in 1mM 4AP illustrates high frequency activity. (C) The frequency of neuronal network activity is significantly increased following 1mM 4AP addition (3300-4200s) when compared to baseline (0-900s) (*P<0.05, n=3 zebrafish, paired Student's t-test). However there is no significant difference in the power of neuronal network events following 1 mM 4AP addition (3300-4200s) when compared to baseline (0- 900s) (P>0.05, n=3 zebrafish, paired Student's t-test).

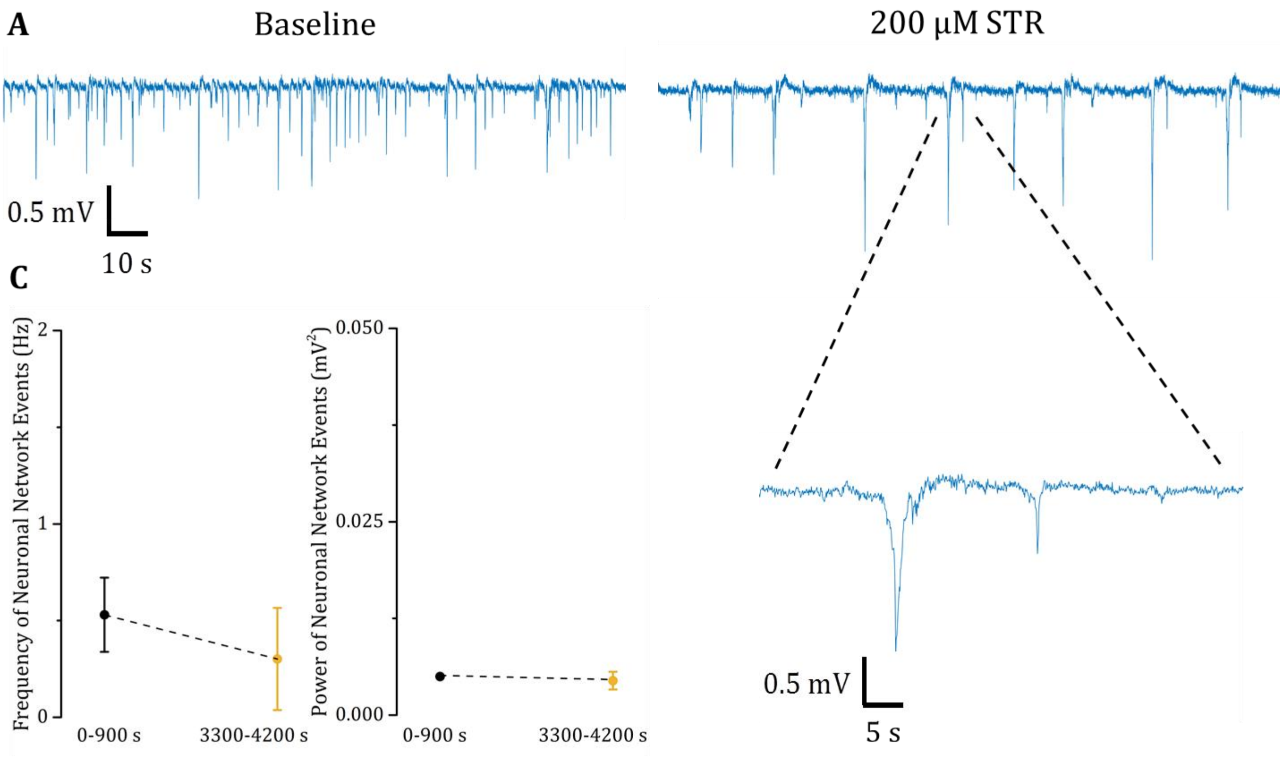

Supplementary Figure 8| 200 μM STR application has no effect on the frequency or power of neuronal network events. Representative trace of baseline and 200μM STR optic tectum waveform activity. (B) Enhanced electrographic activity in 200μM STR demonstrates increased amplitude of neuronal network events compared to baseline. (C) The frequency and power of neuronal network activity in 200μM STR (3300-4200s) is not significantly different compared to baseline (0-900s) (P>0.05, n=2 zebrafish, paired Student's t-test).

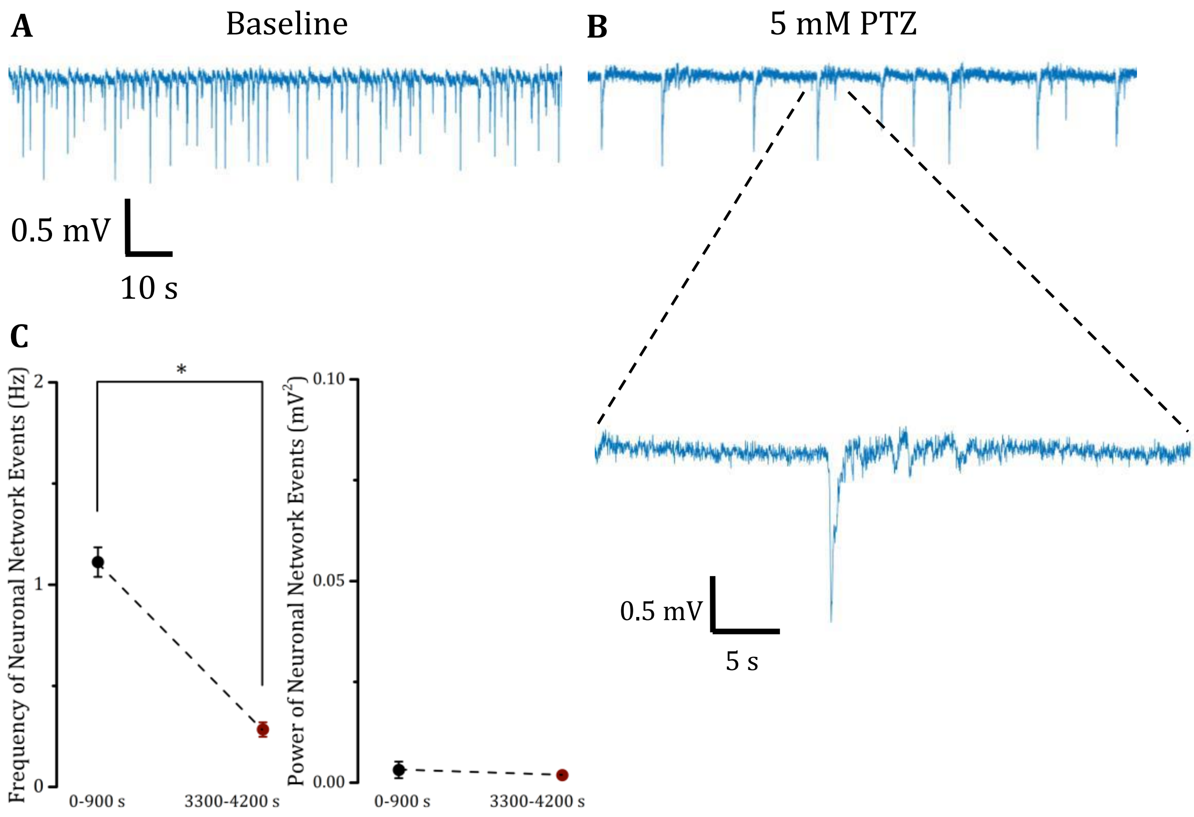

Supplementary Figure 9| 5mM PTZ application shows a decrease in the frequency of neuronal network events. **A)** Representative trace of baseline and 5 mM PTZ optic tectum waveform activity. **B)** Electrographic activity in 5mM PTZ indicates a similar amplitude of neural events with reduced frequency. C) The frequency of neuronal network activity is significantly decreased following 5mM PTZ addition (3300-4200s) when compared to baseline (0-900s) (*P<0.05, n=3 zebrafish, paired Student's t-test). However there is no significant difference in the power of neuronal network events (P>0.05, n=3 zebrafish, paired Student's t-test).

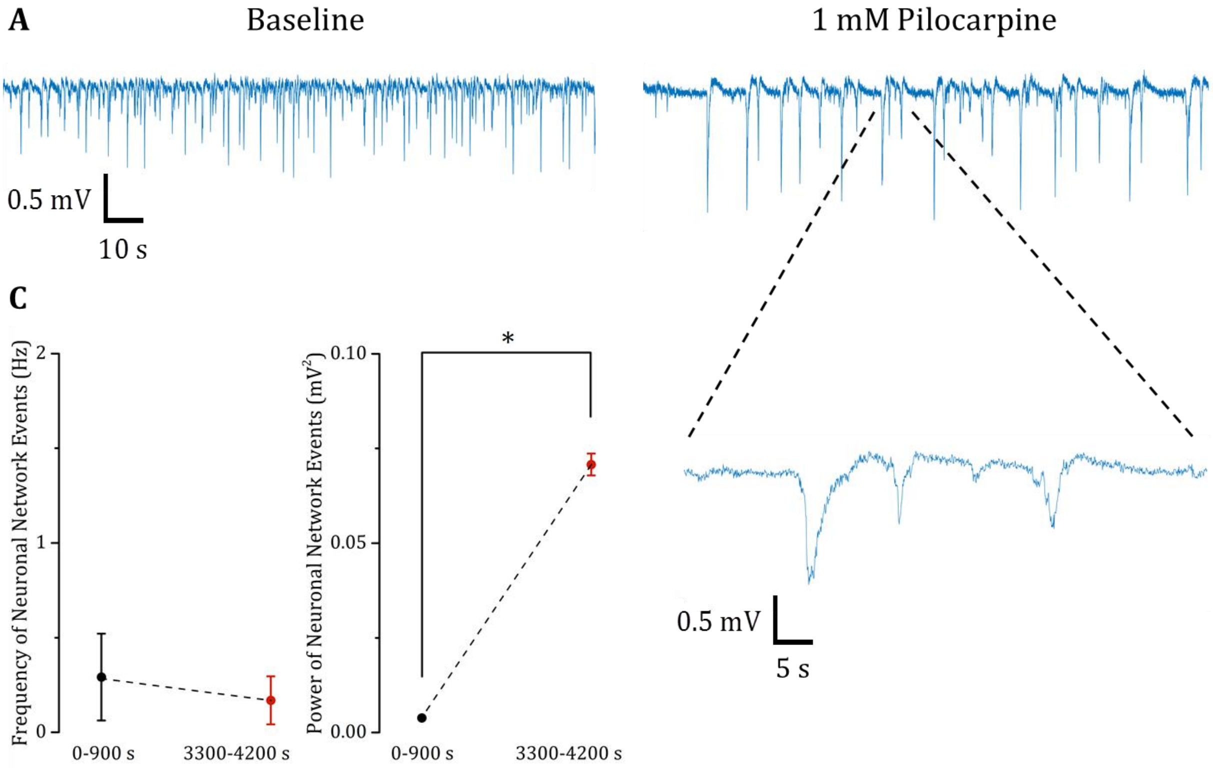

Supplementary Figure 10| 1mM Pilocarpine application shows an increase in the power of neuronal network events. **A)** Representative trace of baseline and 1mM Pilocarpine optic tectum waveform activity. **B)** Electrographic activity in 1mM Pilocarpine indicates an increased power of neural events. **C)** There is no significant change in the frequency of neuronal network activity following 1mM Pilocarpine addition (3300-4200s) when compared to baseline (0-900s) ($P>0.05$, n=2 zebrafish, paired Student's t-test). However there is a significant increase in the power of neuronal network events (*$P<0.05$, n=2 zebrafish, paired Student's t-test).

Supplementary Video 1) Example wide-field video recorded showing the real-time changes in neural activity (as shown by changes in GCaMP6s fluorescence) in a curarized 4dpf *elavl3*:GCaMP6s zebrafish larva with no chemoconvulsant treatment recorded at 10x on a standard inverted microscope under constant 490 nm illumination.

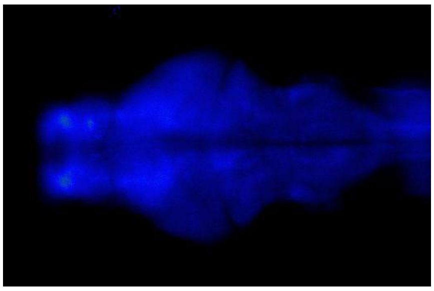

Supplementary Video 2) Example wide-field video recorded showing the real-time changes in neural activity (as shown by changes in GCaMP6s fluorescence) in a curarized 4dpf *elavl3*:GCaMP6s zebrafish larva following treatment with 5mM PTZ, recorded at 10x on a standard inverted microscope under constant 490 nm illumination.

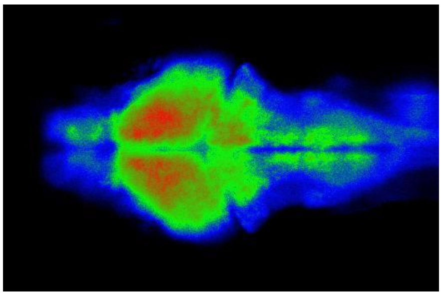

Supplementary Video 3) Example wide-field video recorded showing the real-time changes in neural activity (as shown by changes in GCaMP6s fluorescence) in a curarized 4dpf *elavl3*:GCaMP6s zebrafish larva following treatment with 200µM strychnine, recorded at 10x on a standard inverted microscope under constant 490nm illumination.

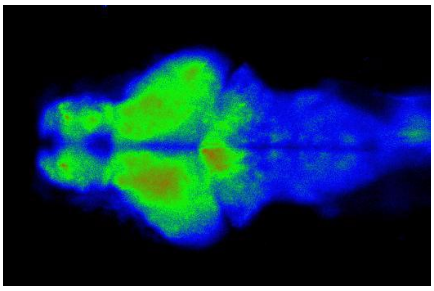

Supplementary Video 4) Example wide-field video recorded video showing the real-time changes in neural activity (as shown by changes in GCaMP6s fluorescence) in a curarized 4dpf *elavl3*:GCaMP6s zebrafish larva following treatment with 1mM 4AP, recorded at 10x on a standard inverted microscope under constant 490nm illumination.

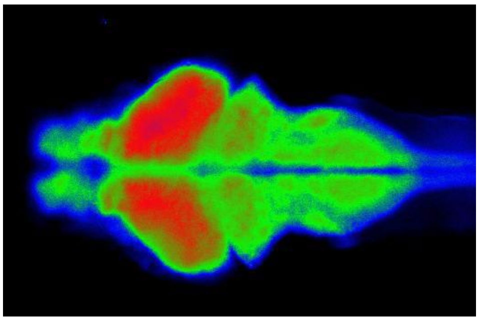

Supplementary Table 6| Summary of the average fluorescence intensity for each broad category of anatomical region, as a % of the average of all categories grouped together in the 4 untreated control fish groups. Data shown are the average of the fluorescence intensity of all fish for each category, within each control group, as a % of the average fluorescence intensity across the whole brain (all 5 categories). The mean and SEM for each of these categories across the 4 control groups are shown in the right most column. In each control group average was taken across 8 animals.

	Untreated control fish group					
**Category**	**1**	**2**	**4**	**4**	**Mean**	**SEM**
**Telencephalon**	34	31	41	52	39	5
**Diencephalon**	101	80	44	74	75	12
**Mesencephalon**	105	119	85	134	111	11
**Rhombencephalon**	116	130	145	115	127	7
**Ganglia**	48	66	62	32	52	8

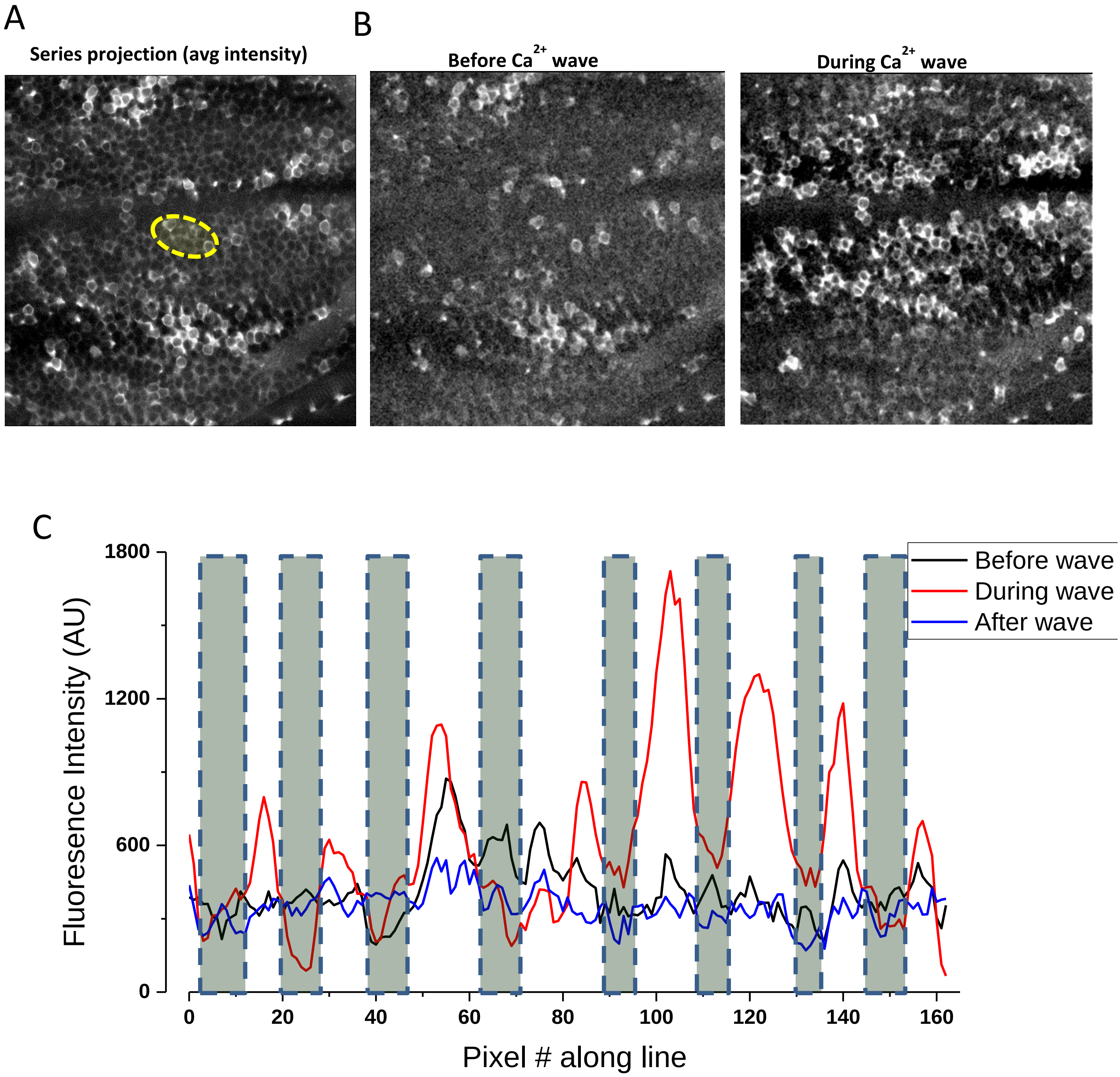

Supplementary Figure 11| Multiphoton timelapse imaging confirms cytoplasmic restriction of GCaMP6s expression in zebrafish neurons. **A)** An average intensity series projection of 99 images collected at 2Hz from a 4AP treated 3dpf zebrafish in vivo using 2 photon imaging (16X Nikon objective 910 nm excitation). Note the tight packing of the neurons and the clear rings of cytoplasmic fluorescence around darker nuclei in almost all cells. The highlighted region was used for the analysis in (C). **B)** Images collected before (left) and during (right) a generalized "wave" of neural activity that lasted around 8s. This was reported by a wide reaching rise in intracellular Ca^{2+} (as indicated by the increased GCaMP6s fluorescence in most cells). **C)** A plot of fluorescence intensity along a line drawn across the diameters of 8 adjacent neurons within the highlighted region in (A). The pixel by pixel intensity plot along the line is shown before (black), during (red) and after (blue) the wave of electrical activity illustrated in (B). The approximate position of each of the 8 nuclei are show by the vertical rectangles, note that the observed increases in Ca^{2+} seen during the wave of activity are largely extranuclear.

Supplementary Video 5) Example video recorded from a multiphoton based assessment of an elavl3:GCaMP6s zebrafish larva following treatment with 1mM 4AP. This video serves to illustrate the level of resolution that can be achieved in this transgenic model, and clearly shows the cytosolic staining of GCaMP6s in each individual neuron.

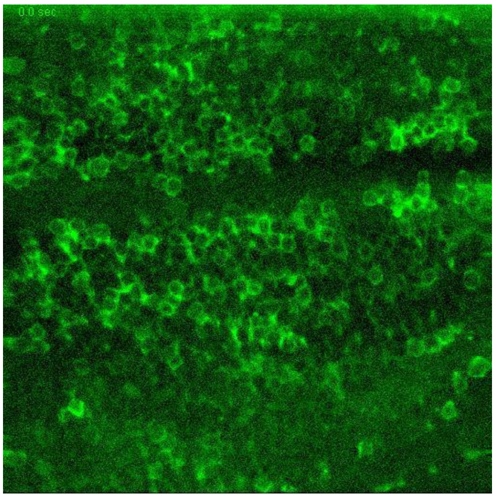

Supplement: Supplementary file 1 — Supplementary information [file 41598_2017_6646_MOESM1_ESM.pdf]
